# *In Silico* Prediction of Inhibition of Promiscuous Breast Cancer Resistance Protein (BCRP/ABCG2)

**DOI:** 10.1371/journal.pone.0090689

**Published:** 2014-03-10

**Authors:** Yi-Lung Ding, Yu-Hsuan Shih, Fu-Yuan Tsai, Max K. Leong

**Affiliations:** 1 Department of Chemistry, National Dong Hwa University, Shoufeng, Hualien, Taiwan; 2 Center for General Education, Chang Gung University, Taoyuan, Taiwan; 3 Department of Life Science and Institute of Biotechnology, National Dong Hwa University, Shoufeng, Hualien, Taiwan; 4 Department of Medical Research and Teaching, Mennonite Christian Hospital, Hualien, Taiwan; Kyushu University, Japan

## Abstract

**Background:**

Breast cancer resistant protein has an essential role in active transport of endogenous substances and xenobiotics across extracellular and intracellular membranes along with P-glycoprotein. It also plays a major role in multiple drug resistance and permeation of blood-brain barrier. Therefore, it is of great importance to derive theoretical models to predict the inhibition of both transporters in the process of drug discovery and development. Hitherto, very limited BCRP inhibition predictive models have been proposed as compared with its P-gp counterpart.

**Methodology/Principal Findings:**

An *in silico* BCRP inhibition model was developed in this study using the pharmacophore ensemble/support vector machine scheme to take into account the promiscuous nature of BCRP. The predictions by the PhE/SVM model were found to be in good agreement with the observed values for those molecules in the training set (*n* = 22, *r* ^2^ = 0.82, 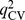  = 0.73, RMSE  =  0.40, *s* = 0.24), test set (*n* = 97, *q* ^2^ = 0.75–0.89, RMSE  = 0.31, *s* = 0.21), and outlier set (*n* = 16, *q* ^2^ = 0.72–0.91, RMSE  =  0.29, *s* = 0.17). When subjected to a variety of statistical validations, the developed PhE/SVM model consistently met the most stringent criteria. A mock test by HIV protease inhibitors also asserted its predictivity.

**Conclusions/Significance:**

It was found that this accurate, fast, and robust PhE/SVM model can be employed to predict the BCRP inhibition of structurally diverse molecules that otherwise cannot be carried out by any other methods in a high-throughput fashion to design therapeutic agents with insignificant drug toxicity and unfavorable drug–drug interactions mediated by BCRP to enhance clinical efficacy and/or circumvent drug resistance.

## Introduction

ATP-binding cassette (ABC) transporters comprise a large super family of transmembrane proteins that utilize the energy of ATP hydrolysis to actively transport a broad range of endogenous substances, such as bile acids, cholesterol, ions, and peptides, across extracellular and intracellular membranes in an ATP-dependent manner [1]. In addition, they also plays a dominant role in detoxification and protection against cytotoxic agents by effluxing xenobiotics from the cells [2]. As such, ABC transporters play a critical role in drug absorption, distribution, metabolism, excretion, and toxicity (ADME/Tox) [3].

Hitherto, 52 human ABC transporters, which can be divided into 7 subfamilies, namely ABCA to ABCG based on sequence similarities, have been identified [4]. Overexpression of ABC transporters can lead to multi-drug resistance (MDR). This can be resulted when susceptible cells or cell lines to a given drug becomes cross-resistant to other co-administrated drugs, *viz*. polypharmacy. In many cases, the emergence of MDR bacterial strains causing gonorrhea, pneumonia, cholera, and tuberculosis appears as the major cause of therapeutic failure [5], [6]. As a result, MDR remains a major obstacle to various kinds of clinical treatment.

In addition to the well-recognized role of P-glycoprotein (P-gp), which is encoded by *MDR1* (*ABCB1*) gene, breast cancer resistance protein (BCRP), which is encoded by *ABCG2* gene or mitoxantrone-resistance (MXR) gene and located on chromosome 7q22 [7], [8], also plays an increasingly important role in producing MDR tumor cells [9]. For instance, the sensitivity of the insulin-like growth factor (IGF) inhibitor BMS-536924 was reduced in MCF-7 cell lines overexpressing BCRP [10]. On the other hand, its sensitivity was restored in BCRP knockdown MCF-7 cell lines [10]. Consequently, the BCRP inhibitors can be expected to be clinically useful. For instance, the sensitivity of mitoxantrone, which is a substrate of BCRP, can be restored by sildenafil, which is a phosphodiesterase type 5 (PDE5) inhibitor for the treatment of erectile dysfunction and pulmonary arterial hypertension [11].

Inhibition of BCRP can lead to adverse drug–drug interactions (DDIs) [12]. For example, it has been observed clinically that loss-of-function variants of *ABCG2* affected the pharmacokinetics and pharmacodynamics (PK/PD) profiles of the cholesterol lowering agent rosuvastatin in Chinese and Caucasian patients [13]-[15]. Therefore, inhibition of BCRP transport function by DDIs should be preferably avoided to minimize drug toxicity [3].

Furthermore, it has been demonstrated that BCRP, P-gp, and multidrug resistance-associated protein 4 (ABCC4/MPR4) are the main ABC transporters responsible for limiting drug transport across the blood-brain barrier (BBB) [16]. For instance, erlotinib, which is an epidermal growth factor receptor (EGFR) tyrosine kinase inhibitor (TKI), can be used for the treatment of non-small cell lung cancer (NSCLC) and pancreatic cancer [17], which are the leading causes of cancer-related mortality in the United States [18]. The BBB permeation of erlotinib can be predominantly limited by BCRP [19], [20], reducing the likelihood of central nervous system (CNS) adverse side-effects. On the other hand, the clinical efficacy of erlotinib for treating patients with metastatic brain cancer from both types of cancer will be restricted by BCRP [21], [22]. Thus, co-administration of BCRP inhibitors may provide a potential therapeutic strategy to improve delivery and efficacy of erlotinib against CNS tumors [23], [24].

To this end, it is of practical importance to find inhibitors of P-gp and BCRP transporters to circumvent MDR or to increase the BBB permeation for CNS therapeutic agents in addition to their pivotal and profound roles in PK/PD [25], [26]. Unfortunately, inhibitors of ABC transporters have little practical applications due to their side effects [27]. It is important to note that the availability of BCRP inhibitors is even more limited relative to those of P-gp counterparts. In fact, there are a variety of molecules that can be transported by both P-gp and BCRP [28], yet development of BCRP-specific inhibitors remains an important task [29].

*In silico* ADME/Tox prediction plays an increasing role in drug discovery and development because of its efficiency, low cost, and throughput [30]. In fact, a number of pharmacophore, CoMFA, and QSAR models have been proposed to predict the inhibition of BCRP [31]–[39] and a brief summary can be found elsewhere [35], [40]. However, BCRP is highly promiscuous *per se* when interacting with a broad spectrum of structurally diverse ligands [41], making it rather difficult to accurately model drug-protein interaction [42]. Such perplexing system, nevertheless, can be resolved using a molecular modeling scheme, devised by Leong [43], in which the pharmacophore ensemble (PhE) was constructed by assembling a group of pharmacophore hypotheses to encode the protein conformational flexibility and multiple ligand orientations in conjunction with support vector machine (SVM) regression. The PhE/SVM scheme is faster and less constraint as compared with any other analog-based modeling schemes [44]. Practically, the PhE/SVM scheme has been employed to accurately model human *ether-á-go-go* related gene (hERG) potassium channel [43], human cytochromes [45], [46], human pregnane X receptor (hPXR) [47], and P-gp transporter [48], which are highly promiscuous proteins *per se* [42]. Herein, this study was aimed specifically at developing an *in silico* model based on the PhE/SVM scheme to accurately and rapidly predict the BCRP inhibition of a broad spectrum of molecules to greatly facilitate drug discovery to design molecules with a better PK/PD profile.

## Materials and Methods

### Data Compilation

The complete data set contains 135 molecules belonging to different structural classes, which were collected from 5 different sources after comprehensive literature search and cautious examinations of their assay conditions [49]–[53]. If there were more than one IC_50_ value for a given molecule and they were in very close range, the averaged value was taken to assure better consistency. Chemical structures without defined stereochemistry such as racemates were excluded from selection. All molecules enrolled in this study and references to the literature are listed in Table S1.

Each molecule in the data set was subjected to conformational search to generate the low-lying conformations using mixed Monte Carlo multiple minimum (MCMM) [54]/low mode [55] in conjunction with the GB/SA hydration algorithm [56] implemented in the *MacroModel* package (Schrödinger, Portland, OR), in which the energy minimization was carried out using the truncated-Newton conjugated gradient method (TNCG) with the selection of MMFFs force field [57], and the solvation effect was taken into consideration using water as solvent with a constant dielectric constant. No more than 255 unique conformations were generated for each compound to maximize the coverage in the conformational space within the energy window of 20 Kcal/mol (or 83.7 KJ/mol) above the global minimum energy conformation in order to be accommodated by *HypoGen* (*vide infra*), which takes into account all conformations for each molecule in training set for pharmacophore hypothesis generation as compared with any other QSAR schemes, which normally employ only the most stable single conformation.

### Pharmacophore Development

The development of PhE/SVM model can be divided into two parts, namely pharmacophore ensemble and SVM regression. The architecture of PhE/SVM scheme can be illustrated by Figure 3 of Chen *et al*. [47]. The automatic pharmacophore generations were carried out using the *HypoGen* module implemented in *DiscoveryStudio* (Accelrys, San Diego, CA). The theory and algorithm of *HypoGen* have been described in detail elsewhere [58]. Basically, *HypoGen* attempts to correlate activities with the spatial arrangement of a variety of chemical features through three phases, namely construction, subtraction, and optimization as compared with any other QSAR techniques [59]. Generated hypotheses, whose chemical features are shared by those most active molecules in the training set, are identified in the constructive phase. Those chemical features are common to the most and the least active compounds are eliminated in the subtractive phase. Finally, small perturbations are applied to those remaining hypotheses and their corresponding scores are evaluated based on the predictive errors as well as the level of complexity in the optimization phase.

As such, the chemical characteristics and their associated activities of those selected samples in the training set predominately determine the predictivity of a generated pharmacophore hypothesis. In other words, both structural diversity and wide coverage of the activity range should be taken into consideration. More specifically, the most active, several moderately active, and some inactive compounds should be included in order to obtain critical information on pharmacophore requirements. Theoretically, an ideal training set should comprise of at least 16 molecules to warrant its statistical significance, 4–5 orders of magnitude in biological activity, approximately equal compounds in each order of magnitude, and novel information concerning structure-activity relationship [58].

Twenty-two molecules, whose IC_50_ values spanned over 4 logarithm units, were deliberately selected from the compound collections to construct the training set for automatic pharmacophore generation and regression after manual scrutinization of structure-activity relationship of all compounds to eliminate any chemical or biological redundancy present in the samples. The remaining ninety-seven molecules from the compound collections with biological activities spanning over about 4 logarithm units were treated as the test set to validate those generated models.

Initially, a number of test runs were carried out to evaluate the selection of those molecules in the training set by selecting all chemical features that have been adopted in previously published pharmacophore models [31]–[34], [37]–[39]. The preliminary calculations revealed the importance of hydrogen bond acceptor (HBA), hydrophobic (HP), and ring aromatic (RA) chemical features, which describe the intermolecular interactions between a highly electronegative atom such as an O, N, or F atom on the ligand and an H atom on the protein, between nonpolar moieties on both ligand and protein, and between the π–systems over aromatic rings on both ligand and protein, respectively. Consequently, those chemical features as well as various combinations of the minimum and maximum numbers of each selected chemical feature, the total number of chemical features, chemical feature weights, and chemical feature tolerances were adopted in order to maximize the hypothesis diversity and performance.

### SVM Calculations

The pIC_50_ values predicted by those pharmacophore hypotheses in the PhE were treated as input for the SVM calculations. As such, the number of pharmacophore models in the ensemble is equivalent to the dimensionality of the SVM input space. The SVM calculations were carried out by the *LIBSVM* package (software available at http://www.csie.ntu.edu.tw/~cjlin/libsvm), which consists of two modules for regression, namely, *svm-train* and *svm-predict*, for producing SVM models based on those samples in the training set and validating those generated models by predicting those molecules in the test set, respectively. The optimal SVM models were automatically yielded using an in-house perl script [60] to systemically scan through those runtime parameters, namely cost *C*, the width of the kernel function *γ*, and *ε* and *ν* in case of *ε*-SVR and *ν*-SVR, respectively.

### Predictive Evaluations

The coefficient for the least squares regression line correlating observed (ordinate) and predicted (abscissa) values (*r* ^2^) was calculated according to the following equation:
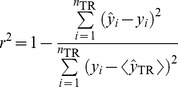
(1)where 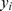
and 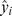 are the observed and predicted values, respectively; and 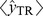 and *n*_TR_ are the mean of predicted values and the number of samples in the training set, respectively. Similarly, the correlation coefficient 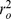 and slope *k* were calculated from the regression line correlating observed (ordinate) and predicted (abscissa) values through the origin, respectively, and 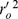 was derived from the regression line correlating predicted (ordinate) and observed (abscissa) values through the origin. Normally, a derived model can be internally validated by *n*-fold cross-validation, which is carried out by randomly dividing the samples into *n* groups and each group being iteratively excluded once, whose activities are then predicted by the model derived from the remaining samples [61]. The developed SVM models were further subjected to internal validation using the 10-fold cross-validation scheme, which was proven to perform better than the widely used leave-one-out [62]. Similar to *r* ^2^, the correlation coefficient of 10-fold cross validation 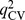 was computed based on the prediction of the leave-out samples.

Furthermore, various modified versions of *r* ^2^ proposed by Roy *et al*. [63] were also evaluated.
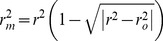
(2)

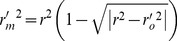
(3)

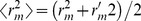
(4)

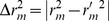
(5)


When applied to the external data set, *viz*. any data set except the training set, the predictive model was subjected to evaluations by the correlation coefficients 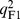, 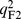, and 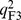 and concordance correlation coefficient (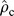), which were proposed by Golbraikh *et al*. [64], Schüürmann *et al*. [65], Consonni *et al*. [61], and Chirico and Gramatica [66].
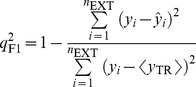
(6)

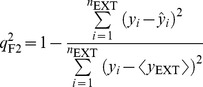
(7)

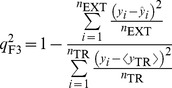
(8)

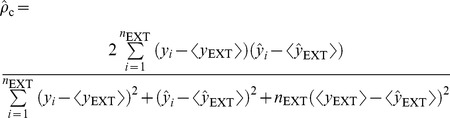
(9)where 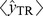 and 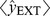 are the averages of predicted values in the training set and external set, respectively, 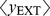 is the mean of observed values in the external set, and *n*_TR_ and *n*_EXT_ are the number of samples in the training set and external set, respectively. In fact, 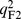, which has been adopted by Organization for Economic Co-operation and Development (OECD) for evaluating the external predictivity of QSAR models, is similar to *r* ^2^ except that the former is applied to the external data set whereas the later is applied to the training set [65].

Finally, the predictivity of all developed models were subjected to evaluations by the most stringent criteria proposed by Golbraikh *et al*. [64], Ojha *et al*. [67], Roy *et al*. [63], and Chirico and Gramatica [66].
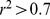
(10)

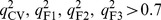
(11)

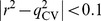
(12)

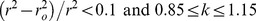
(13)

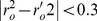
(14)

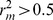
(15)

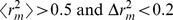
(16)

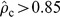
(17)where *r* in equations (12)–(16) represents *r* and *q*_F2_ in the training set and external sets, respectively.

## Results

### PhE

A great number of theoretical models were produced using various selections of chemical features and runtime parameters throughout the automatic pharmacophore generation procedure. Three pharmacophore hypotheses, designated by Hypo A, Hypo B, and Hypo C (listed in File S1), were enlisted to build PhE based on their prediction performances on every individual molecule, which are listed in Table S1, and statistical assessments in the training set and test set, which are summarized in Tables 1 and 2, respectively.

**Table 1:** Statistic parameters correlation coefficient (*r* ^2^), maximum residual (Δ_Max_), mean absolute error (MAE), standard deviation of residual (*s*), RMSE, and cross-validation coefficient 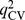 evaluated by Hypo A, Hypo B, Hypo C, and PhE/SVM in the training set.

	Hypo A	Hypo B	Hypo C	PhE/SVM
*r^2^*	0.69	0.67	0.59	0.82
Δ_Max_	1.26	1.08	1.12	0.87
MAE	0.42	0.42	0.50	0.33
*s*	0.31	0.34	0.34	0.24
RMSE	0.52	0.54	0.60	0.40
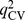	N/A†	N/A	N/A	0.73

† Not applicable.

**Table 2:** Statistic parameters correlation coefficients 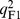, 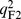, and 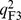, concordance correlation coefficient (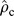), maximum residual (Δ_Max_), mean absolute error (MAE), standard deviation of residual (*s*), and RMSE evaluated by Hypo A, Hypo B, Hypo C, and PhE/SVM in the test set.

	Hypo A	Hypo B	Hypo C	PhE/SVM
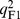	0.45	0.63	0.54	0.75
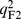	0.45	0.63	0.54	0.75
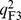	0.76	0.84	0.80	0.89
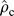	0.69	0.78	0.71	0.86
Δ_Max_	1.30	1.50	1.55	0.88
MAE	0.31	0.21	0.29	0.23
*s*	0.34	0.31	0.31	0.21
RMSE	0.46	0.37	0.42	0.31

**Figure 1:**
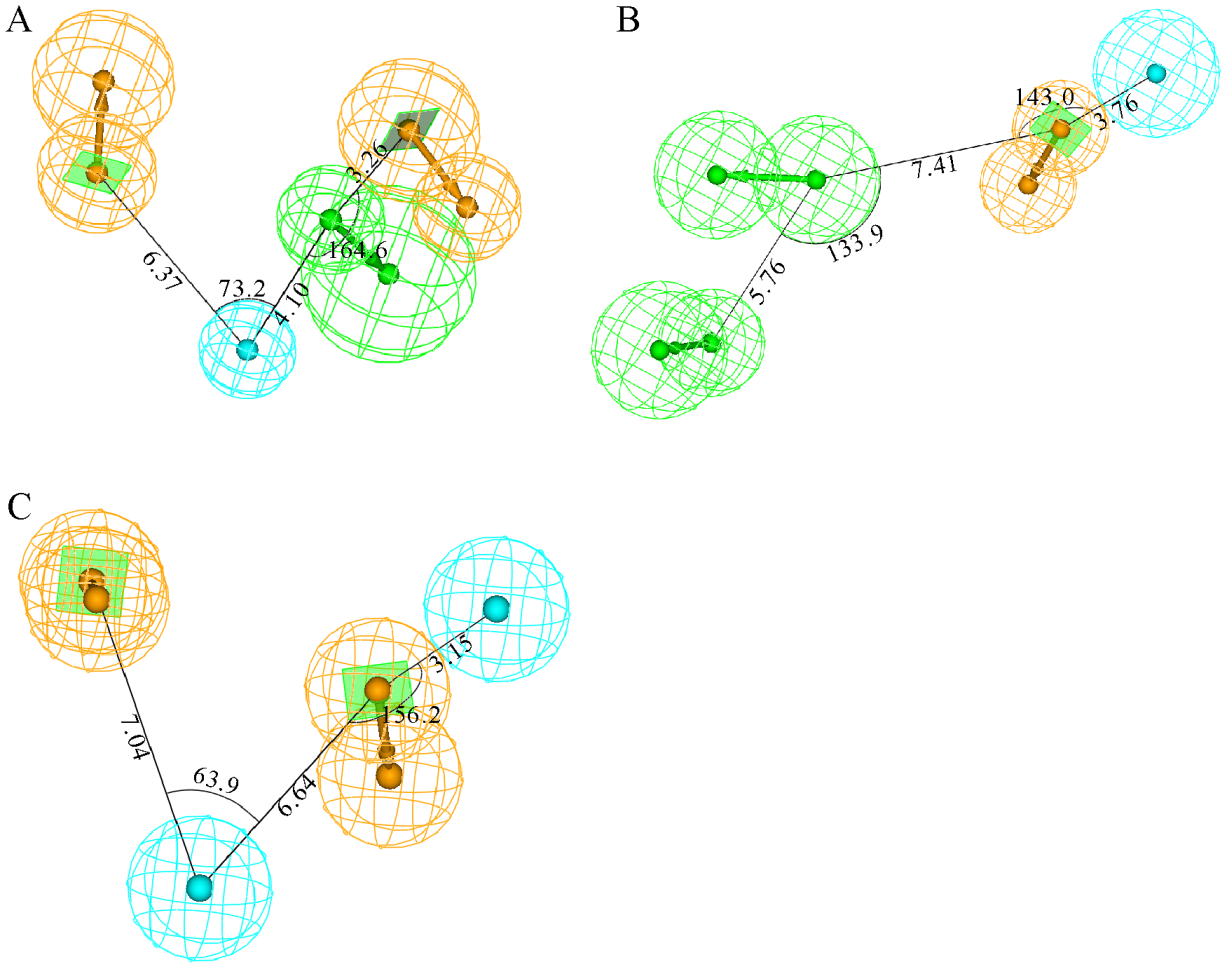
Pharmacophore models in the ensemble. Generated pharmacophore models (A) Hypo A, (B) Hypo B, and (C) Hypo C, consisting of hydrogen-bond acceptor (green), hydrophobic (light blue), and ring aromatic (orange) chemical features. The interfeature distances and angles among features, depicted in white, are measured in Ångstroms and degrees, respectively.

**Figure 2:**
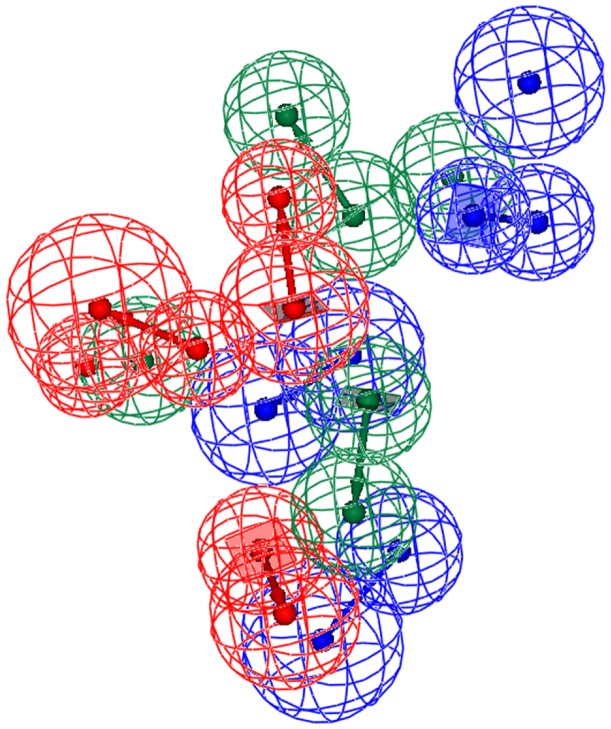
Superposed pharmacophore models. Superposition of three pharmacophore models Hypo A, Hypo B, and Hypo C, denoted in red, blue, and green, respectively.

These three pharmacophore hypotheses are comprised of different combinations of chemical features, namely one HBA, one HP, and two RAs in Hypo A; two HBAs, one HP, and one RA in Hypo B; and two HPs and two RAs in Hypo C as illustrated by Figure 1, from which it can be observed that they also display different spatial relationships. For instance, both HP and RA are the common features among these three pharmacophore models, and the shortest distances between them are 6.37 Å, 3.76 Å, and 3.15 Å in Hypo A, Hypo B, and Hypo C, respectively. The discrepancies in the relative relationships and absolute topological arrangements among these three theoretical models can be further illustrated by their superposition as shown in Figure 2.

**Figure 3:**
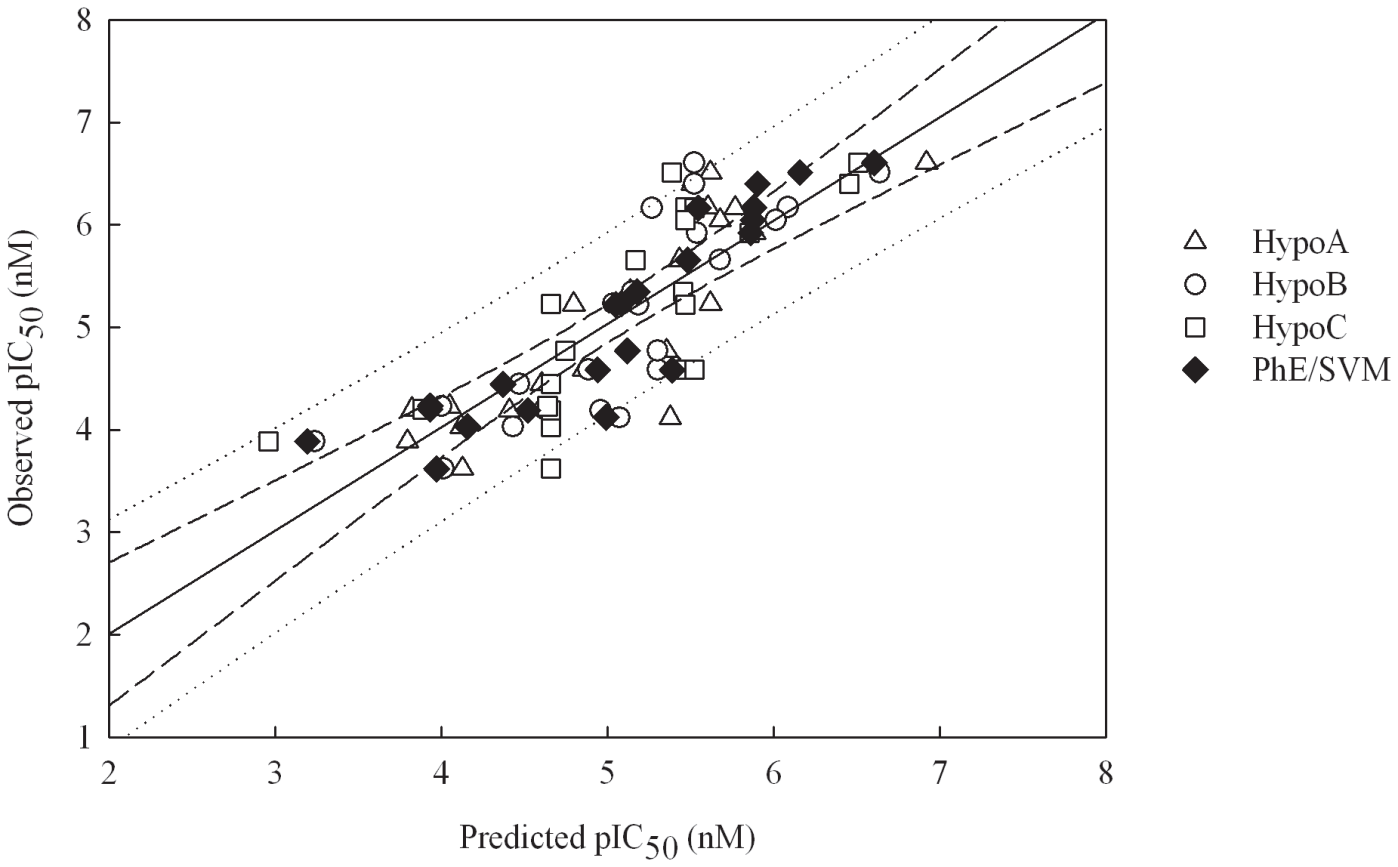
Observed *vs*. predicted pIC_50_ values in the training set. Observed pIC_50_ vs. the pIC_50_ predicted by Hypo A, Hypo B, Hypo C, and PhE/SVM for those molecules in the training set. The solid line, dashed lines, and dotted lines correspond to the PhE/SVM regression of the data, 95% confidence interval for the PhE/SVM regression, and 95% confidence interval for the prediction, respectively.

It can be observed from Figure 3, which displays the scatter plot of observed *vs*. predicted pIC_50_ values for all molecules in the training set, that the predictions by Hypo A, Hypo B, and Hypo C are generally in agreement with observed values in the training set. As such, they produced *r* ^2^ values around 0.60 (Table 1), suggesting that they are of modest statistical significance, which can be further confirmed by their moderate corresponding residuals (Table S1) as well as statistical assessments, namely RMSE, MAE, and *s* (Table 1).

**Figure 4:**
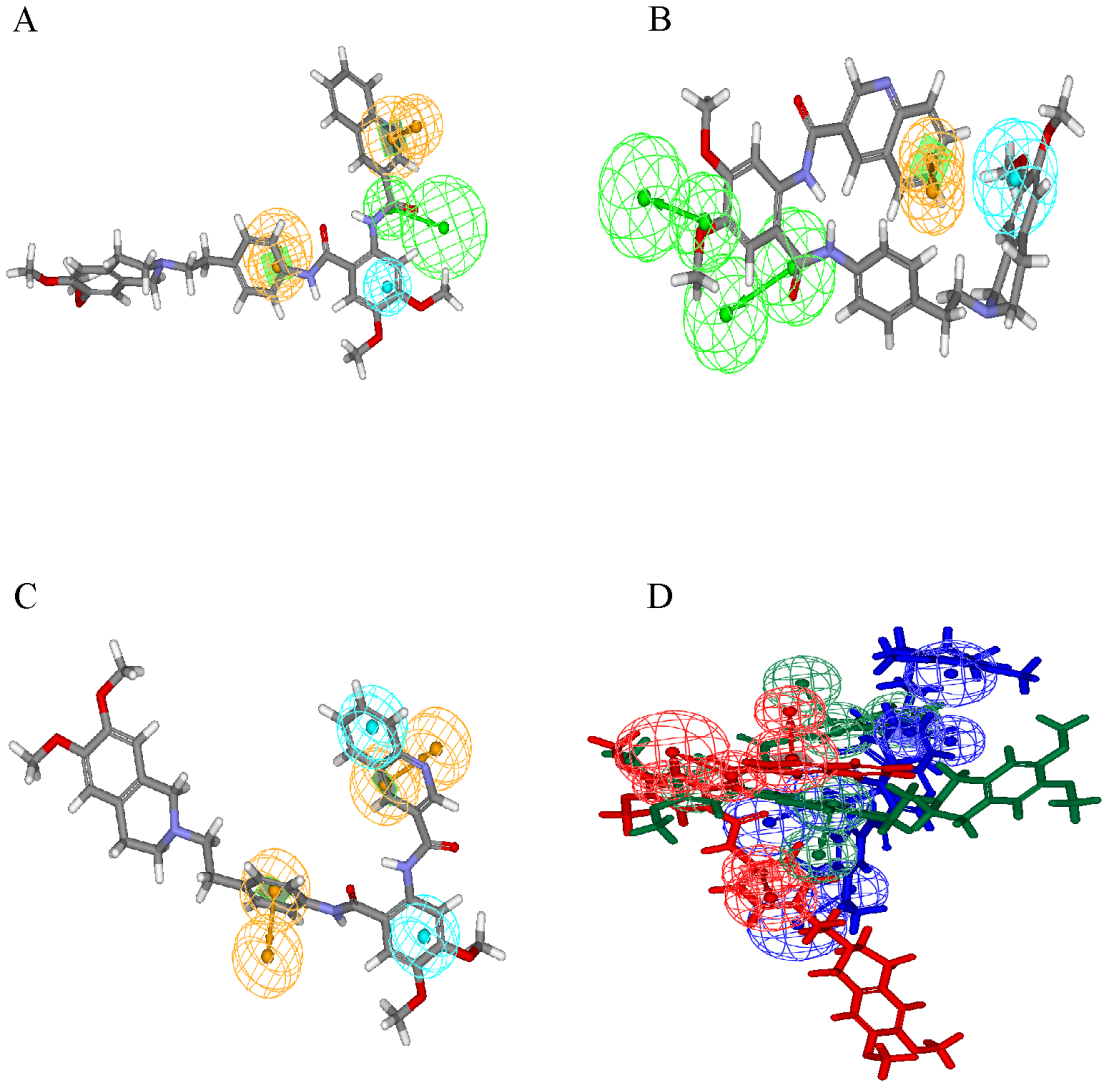
Superposition of pharmacophore models and 22. Pharmacophore models (A) Hypo A, (B) Hypo B, and (C) Hypo C fitted to **22** and (D) overlay of these three models, which are color-coded by red, blue, and green, respectively. The chemical features are described in Figure 1.

Hypo A yielded the maximum residual in the training set when predicting **11** with an absolute value of 1.26, whereas Hypo B and Hypo C generated absolute deviations of 0.95 and 0.54, respectively (Table S1). The maximum residual produced by Hypo B in the training set was resulted from the prediction of **8** with an absolute value of 1.08, yet those absolute errors by Hypo A and Hypo C were only 0.32 and 0.09, respectively. The prediction of **9** by Hypo C deviated most from the observed value with an absolute residual of 1.12, whereas Hypo A and Hypo B showed absolute deviations of 0.89 and 0.13, respectively. Furthermore, the predictions of **5** by Hypo A, Hypo B, and Hypo C only yielded absolute deviations of 0.21, 0.20, and 0.11, respectively. Such discrepancies among these three pharmacophore hypotheses can be further manifested by the predictions of **22** by Hypo A, Hypo B, and Hypo C, in which these three theoretical models interacted with BCRP using different conformations, yielding modest absolute errors of 0.03, 0.38, and 0.07, respectively, as demonstrated in Figure 4A, B, and C. This difference becomes more obvious by the overlay of these three conformations as illustrated in Figure 4D, suggesting the necessity of constructing a PhE to take into account the conformational variations of BCRP.

**Figure 5:**
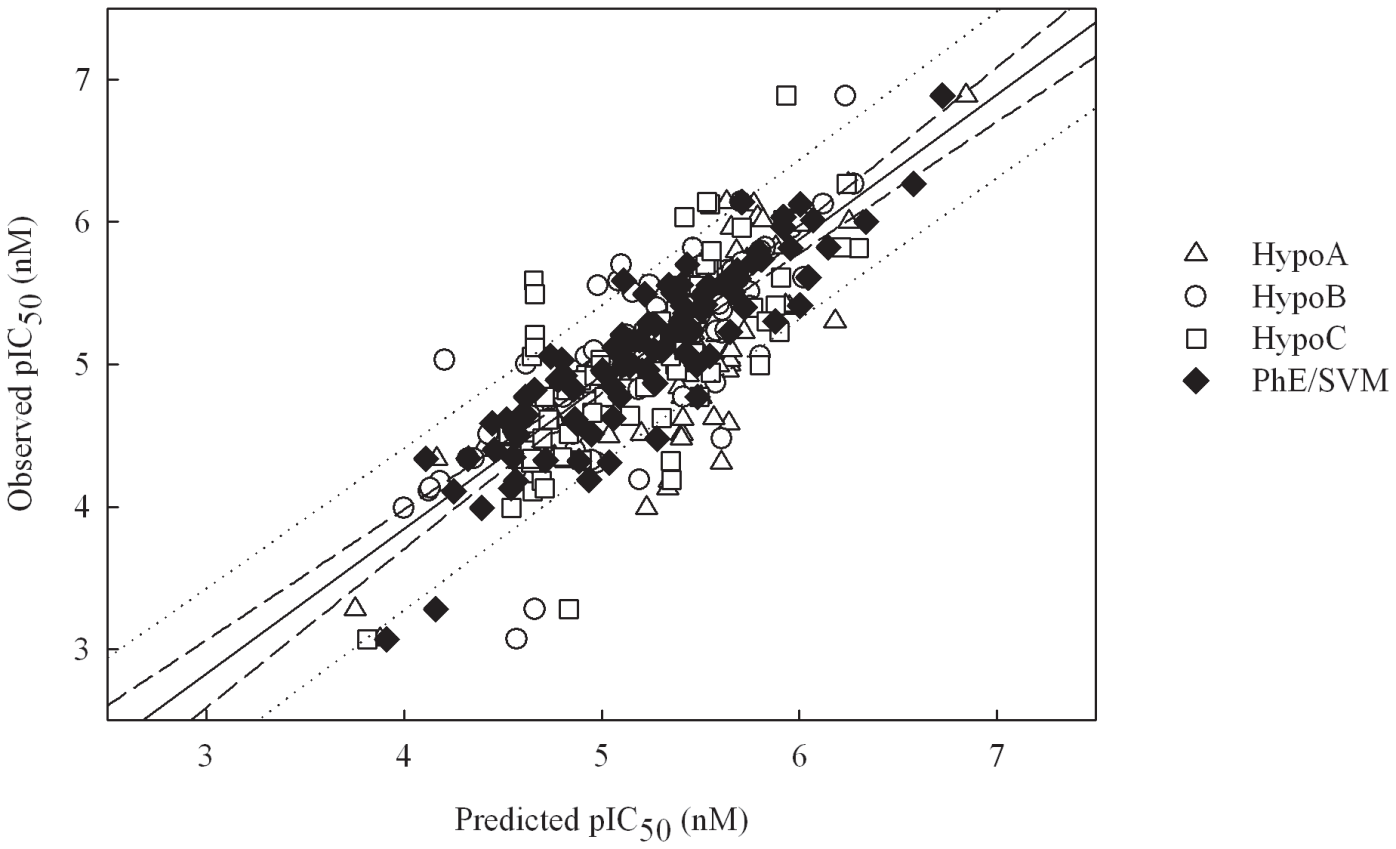
Observed *vs*. predicted pIC_50_ values in the test set. Observed pIC_50_ *vs*. the pIC_50_ predicted by Hypo A, Hypo B, Hypo C, and PhE/SVM for those molecules in the test set. The solid line, dashed lines, and dotted lines correspond to the PhE/SVM regression of the data, 95% confidence interval for the PhE/SVM regression, and 95% confidence interval for the prediction, respectively.

Generally, the predictions by Hypo A, Hypo B, and Hypo C are in agreement with observed values for those molecules in the test set as shown in Table S1 and Figure 5, which exhibits the scatter plot of observed *vs*. predicted pIC_50_ values for all molecules in the test set. In addition, most of statistical evaluations listed in Table 2 also indicate their reasonable performances in the test set. For example, the differences between 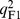 and *r* ^2^ and between 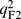 and *r* ^2^ calculated by Hypo B and Hypo C are very small, suggesting their performance consistency in both data sets. Nevertheless, the parameters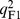 and 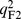 yielded by Hypo A in the test set were reduced by 0.24 from *r* ^2^ in the training set, depicting the fact that Hypo A is a statistically over-trained model. Conversely, some other statistical assessments suggest otherwise. For instance, Hypo A, Hypo B, and Hypo C produced the 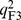 values of 0.76, 0.84, and 0.80 in the test set, respectively, which were larger than their *r* ^2^ values, and their RMSE values unanimously decreased from the training set to the test set. In general, these three theoretical models did not show substantial performance decreases when applied to those molecules in the test set. In addition, the discrepancies among these three pharmacophore models that were observed in the training set can also be found in the test set. For example, Hypo A gave rise to the maximum residual resulted from the prediction of **74** with an absolute value of 1.30 in the test set, whereas Hypo B and Hypo C only produced absolute errors of 0.66 and 0.33, respectively.

### PhE/SVM

The optimal PhE/SVM was generated by assembling those pharmacophore hypotheses in the ensemble, *viz*. Hypo A, Hypo B, and Hypo C, which were further subjected to the SVM regression. The runtime conditions, which were selected based on the predictions of all molecules in the training set and cross-validation, are summarized in Table 3. It can be observed from the scatter plot of observed *vs.* predicted pIC_50_ values shown in Figure 3 that PhE/SVM produced residuals, which are smaller than the maximum deviations yielded by those pharmacophore models in the PhE for most of molecules in the training set, and even the smallest in some cases. For instance, Hypo A, Hypo B, Hypo C, and PhE/SVM gave rise to absolute deviations of 0.39, 0.19, 0.57, and 0.15, respectively, when predicting **1**. As such, PhE/SVM generated the largest *r* ^2^ value and the smallest Δ_Max_, MAE, *s*, and RMSE (Table 1) relative to its counterparts in the PhE, suggesting that PhE/SVM performed better than all of those individual hypotheses in the ensemble in the training set.

**Table 3:** Optimal runtime parameters for the SVM model.

Parameter	Value
SVM type	*ε*-SVR
Kernel type	Radial basis function
*γ*	0.001953131
Cost	263
*ε*	0.35

The generated PhE/SVM was further subjected to 10-fold cross-validation, resulting in the correlation coefficient 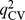 of 0.73 as compared with its *r* ^2^ of 0.82. The large values of both parameters and small difference between them indicate that this PhE/SVM model shows highly statistical significance between the predicted and observed data and it is highly possible that this SVM model is an authentic model statistically.

Furthermore, little decrease in performance was observed when PhE/SVM was applied to those molecules in the test set as manifested by those statistic evaluations listed in Table 2. For example, 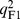 and 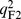 only dropped from *r* ^2^ by 0.07; and both 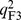 and 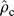 were even higher than *r* ^2^. In addition to those *q* ^2^ parameters, the other statistic variables did not show substantial variations from the training set to the test set, suggesting that PhE/SVM is a statistically consistent predictor since it will otherwise give rise to at least one substantial difference in case of overtraining. More importantly, PhE/SVM also performed better than any of pharmacophore models in the ensemble for those molecules in the test set since PhE/SVM produced the highest 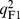, 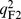, 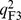, and 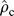 values and the lowest Δ_Max_, MAE, *s*, and RMSE values, except MAE, which was 0.21 produced by Hypo B as compared with PhE/SVM (0.23).

### Robustness Evaluation

**Figure 6:**
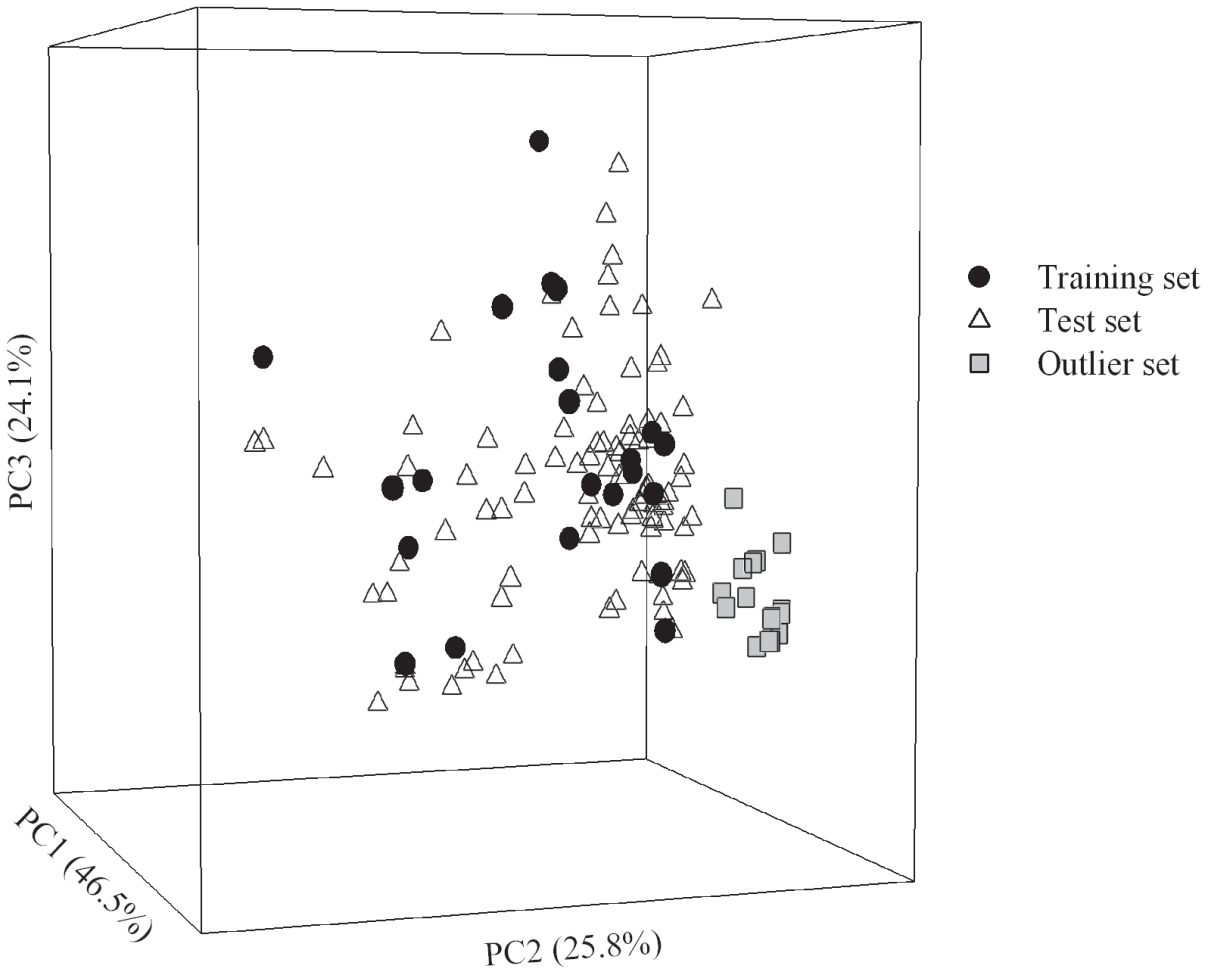
Sample distribution in the chemical space. Molecular distribution for those samples in the training set (blue circle), test set (green triangle), and outlier set (red square) in the chemical space spanned by three principal components.

In general, it is of critical importance to detect those outliers from the sample collections and remove them from model development [68]. Nevertheless, of all adopted molecules in this investigation, 16 molecules were intentionally selected as the outliers to further challenge the extrapolation capacity of developed models. The chemical similarity or dissimilarity can be examined by inspecting the chemical space, which can be constructed by principal component analysis (PCA) [69]. In addition, it has been suggested that outliers can be detected by checking their distributions in the chemical space [68]. To investigate the chemical distinctions between those samples in the outlier set and training set, the molecular descriptors of all molecules adopted in this study were calculated by *DiscoveryStudio* and *E-Dragon* (available at the web site http://www.vcclab.org/lab/edragon/), followed by PCA calculations implemented in *DiscoveryStudio*. Then, all molecules were further projected into a three-dimensional space constructed by the first three principal components (PCs), which explain 96.4% of the variance in the original data, as illustrated by Figure 6. In fact, it can be found by checking their chemical structures that those outliers with no more than two methoxy groups do not contain any carbonyl, nitro, trifluoromethyl, and oxoheterocyclic functionality, in contrast to those training samples. As such, those sample in the outlier set are completely positioned outside the periphery of the training set, indicating their high level of dissimilarity and serving as a good metric for evaluating the robustness of a predictive model [70].

**Figure 7:**
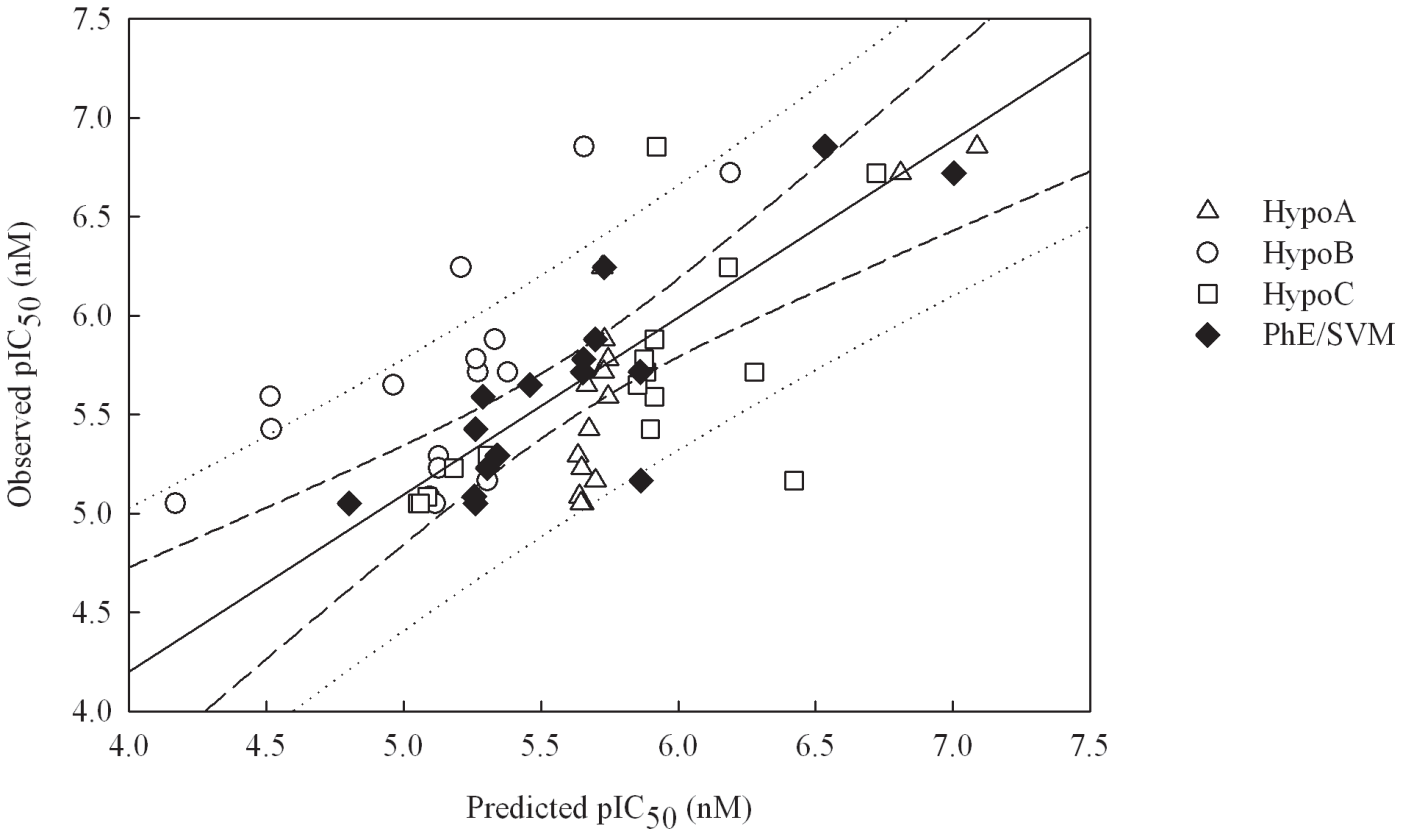
Observed *vs*. predicted pIC_50_ values in the outlier set. Observed pIC_50_ *vs*. the pIC_50_ predicted by Hypo A, Hypo B, Hypo C, and PhE/SVM for those molecules in the outlier set. The solid line, dashed lines, and dotted lines correspond to the PhE/SVM regression of the data, 95% confidence interval for the PhE/SVM regression, and 95% confidence interval for the prediction, respectively.

The prediction results in the outlier set and their associated statistical evaluations are list in Tables S1 and 4, respectively, and the corresponding scatter plot is displayed in Figure 7. The predictions by PhE/SVM are consistent with observed values for all molecules in the outlier set as manifested by Δ_Max_ (0.70), MAE (0.23), *s* (0.17), and RMSE (0.29), which are smaller than their counterparts in the training set. Furthermore, the parameters 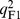, 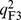, and 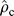 are even larger than *r* ^2^, suggesting that PhE/SVM performed better in the outlier set than in the training set and test set. This presumably can be due to the fact that those designated outliers only spanned over 2 logarithm units. In addition, the computed 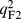 (0.72) is smaller than *r* ^2^ and similar to 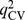. Thus, it can be found that PhE/SVM performed well even when applied to structurally distinct molecules, suggesting that those outliers did not influence the performance of PhE/SVM and PhE/SVM is very robust, which is an important characteristic for a predictive model in practical application.

**Table 4:** Statistic parameters correlation coefficients 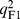, 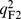, and 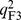, concordance correlation coefficient (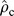), maximum residual (Δ_Max_), mean absolute error (MAE), standard deviation of residual (*s*), and RMSE evaluated by PhE/SVM in the outlier set.

	PhE/SVM
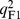	0.87
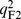	0.72
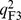	0.91
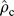	0.85
Δ_Max_	0.70
MAE	0.23
*s*	0.17
RMSE	0.29

### Predictive Evaluations

**Figure 8:**
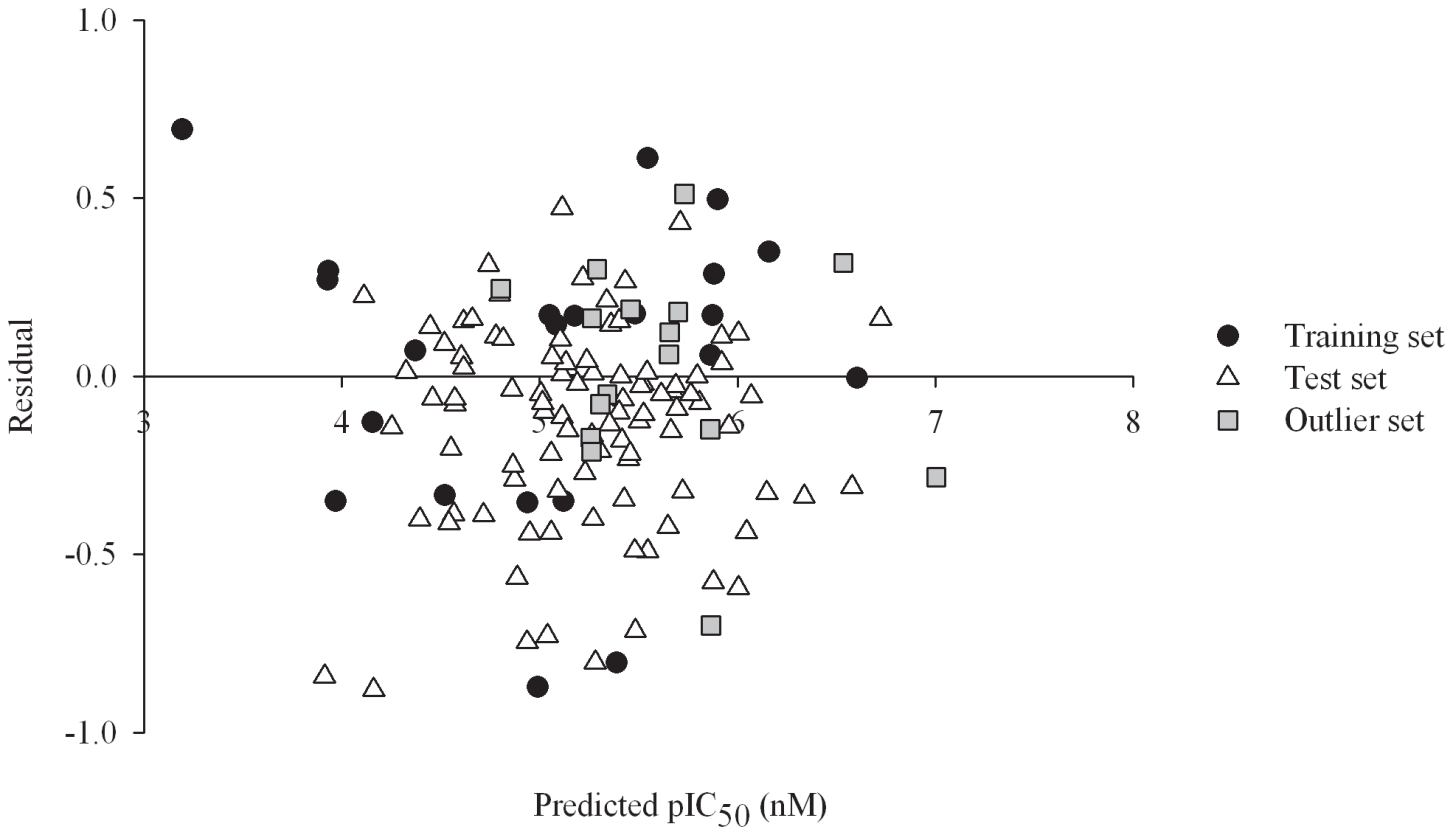
Residual *vs*. predicted pIC_50_ values. Residual *vs*. the pIC_50_ predicted by PhE/SVM in the training set (filled circles), test set (open triangles), and outlier set (gray squares).

It can be found from the scatter plot of the residual *vs*. the pIC_50_ values predicted by PhE/SVM for all of molecules in the training set, test set, and outlier set (Figure 8) that the residuals are approximately symmetrical to the axis of pIC_50_. As such, PhE/SVM gave rise to the mean residuals of 0.03, −0.13, and −0.04 in the training set, test set, and outlier set, respectively (Table S1). These negligible values indicate that there is no systematic bias associated with this PhE/SVM model.

When evaluated by those validation criteria proposed by Golbraikh *et al*. [64], Ojha *et al*. [67], Roy *et al*. [63], and Chirico and Gramatica [66], PhE/SVM showed very high level of predictivity in the training set, test set, and even outlier set that can be manifested by those parameters and assessments listed in Table 5. For instance, PhE/SVM can fulfill the requirements of 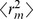 as well as 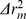, which were considered by Roy *et al*. to be the best validation parameters [63]. Chirico and Gramatica, nevertheless, postulated that both 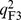 and 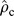 are the most stringent metrics to gauge the predictivity and they have even raised the threshold of all *q* ^2^ parameters from 0.60 to 0.70 [66]. Despite of those facts, PhE/SVM still met those strict assessments. Accordingly, it can be affirmed that this PhE/SVM is a very accurate, precise, and robust predictive model regardless of the chemotypes.

**Table 5:** Validation verification of PhE/SVM based on prediction performance of those molecules in the training set, test set, and outlier set.

	Training set	Test set	Outlier set
*n*	22	97	16
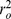	0.84	0.80	0.80
*k*	0.99	0.96	0.99
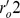	0.79	0.75	0.78
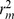	0.70	0.74	0.54
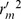	0.68	0.62	0.58
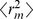	0.69	0.68	0.56
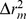	0.02	0.12	0.04
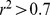	x	N/A	N/A
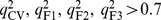	x	x	x
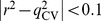	x	x	x
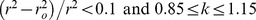	x	x	x
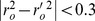	x	x	x
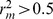	x	x	x
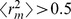 and 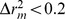	x	x	x
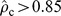	N/A†	x	x

† Not applicable.

### Mock Test

**Figure 9:**
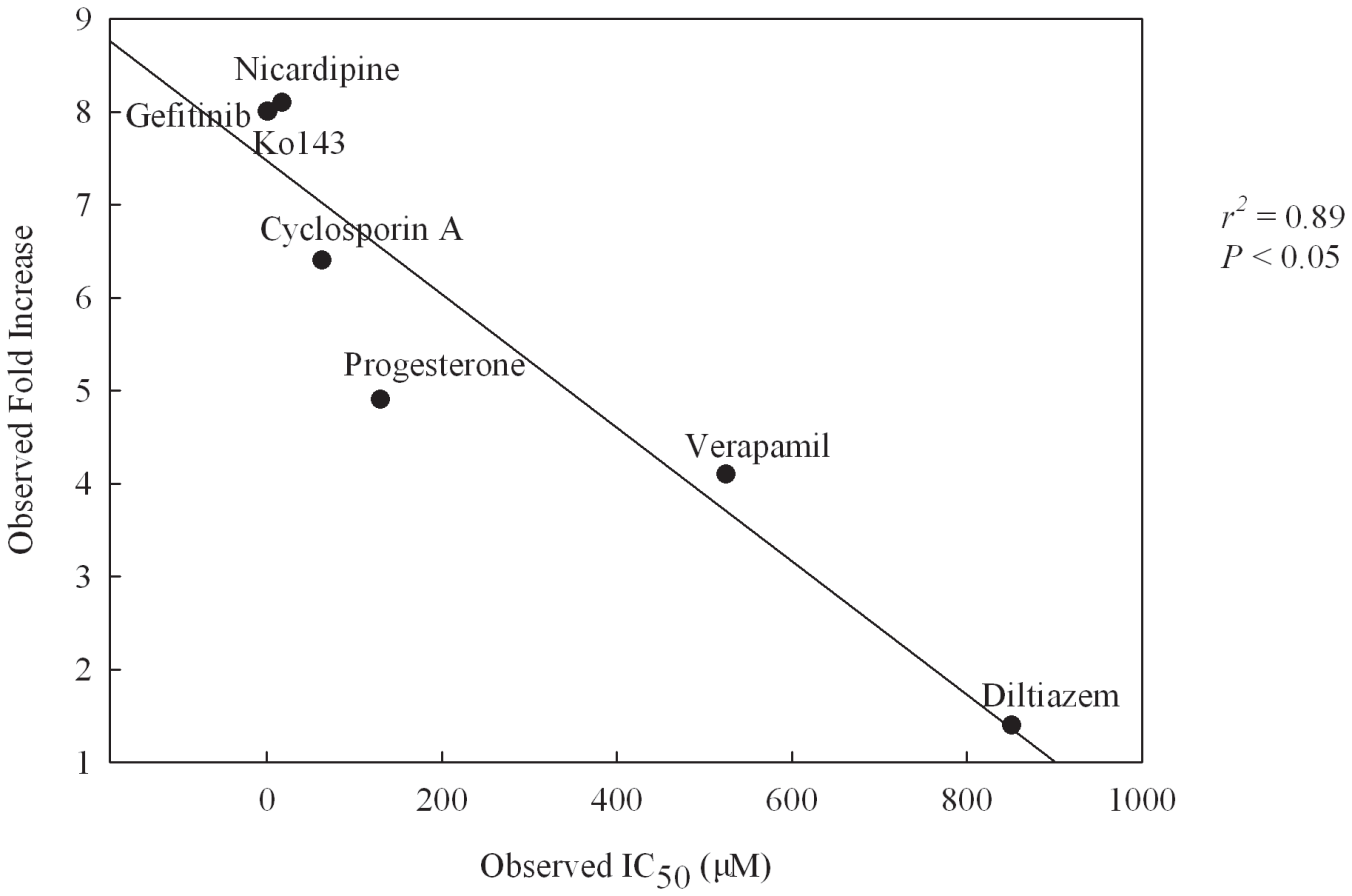
Observed fold increase *vs*. observed IC_50_. Experimental fold increase of BCRP inhibition measured in Saos-2 cells *vs*. observed IC_50_ measured in MCF-7 MX cells.

The derived PhE/SVM was further subjected to test by a number of human immunodeficiency virus (HIV) protease inhibitors (PIs) assayed by Matsson *et al*. [33] in order to mimic the real-world application since HIV PIs are effective BCRP inhibitors but not substrates [71], [72]. Of all molecules assayed by Matsson *et al*. [33], seven were also selected in this study and their names are given in Figure 9, giving rise to a good way to calibrate the testing system. Furthermore, those four HIV PIs were not adopted in this study, representing a solid mock test. Nevertheless, those molecules were assayed using Saos-2 cells to measure fold increase with respect to Ko143, whereas all of compounds enlisted in this investigation were assayed using MCF-7 MX cells to obtain IC_50_ values. To eliminate the inconsistency, the linear correlation between both assay systems for those common molecules was first inspected and the obtained scatter plot is illustrated in Figure 9, from which it can be observed that the experimental values in both systems were highly correlated with each other with an *r* ^2^ of 0.89, suggesting that there is no significant discrepancy in both systems. Thus, it is plausible to examine the PhE/SVM model with those molecules assayed by Matsson *et al.* [33].

**Figure 10:**
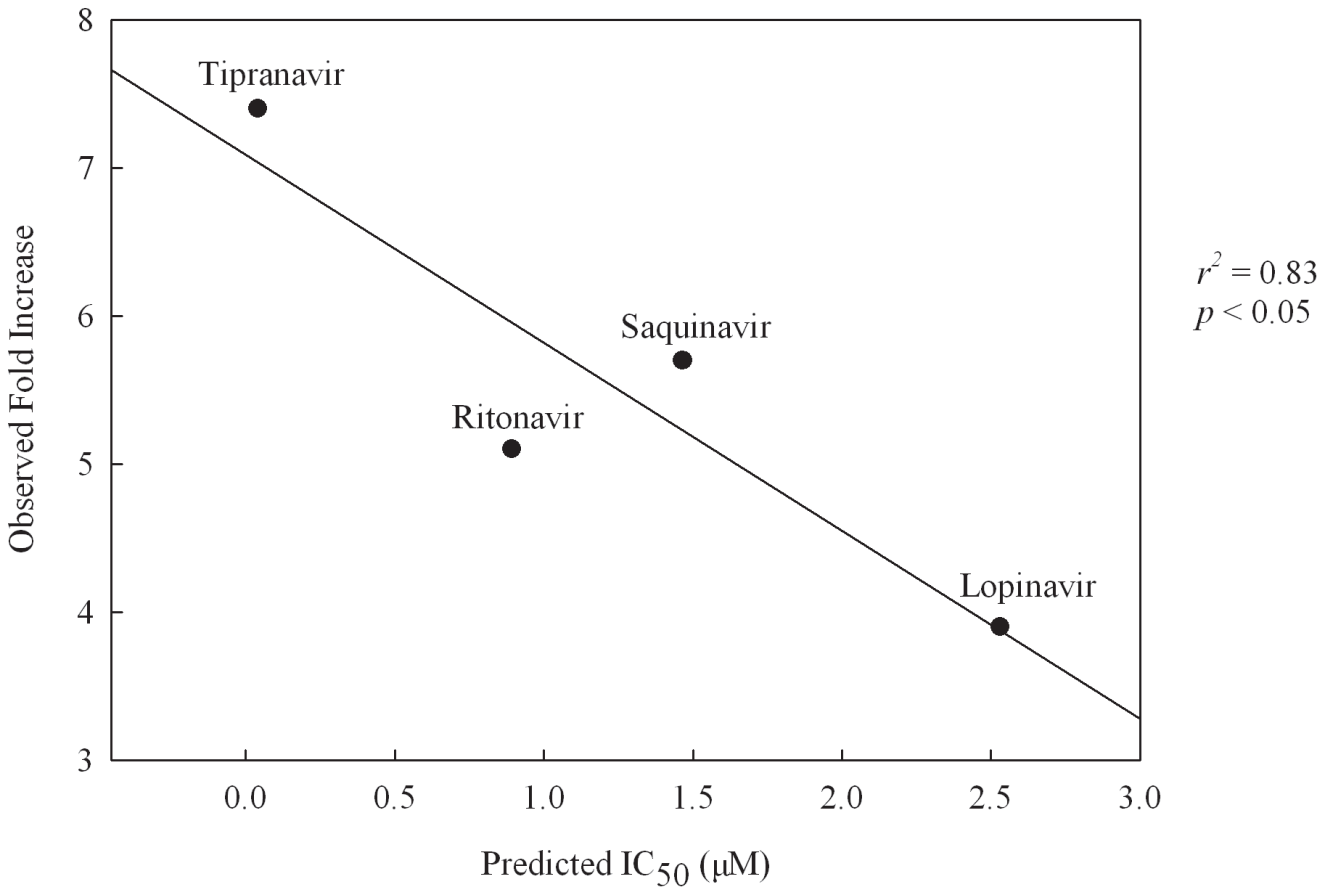
Observed fold increase *vs*. predicted IC_50_. Experimental fold increase *vs*. the IC_50_ predicted by PhE/SVM for those HIV protease inhibitors.

The tested results with those four HIV PIs illustrated in Figure 10, which displays the scatter plot of experimental fold increase *vs*. predicted IC_50_ along with their molecular names, gave rise to an *r* ^2^ value of 0.83 between both systems. The negligible difference between both numbers (0.89 *vs*. 0.83) suggests that the predictions by the PhE/SVM model can almost reproduce the experimental observations and this mock test by HIV PIs unambiguously affirmed the predictive capability of PhE/SVM.

## Discussion

To date, there is only limited number of pharmacophore hypotheses that have been proposed to predict the BCRP inhibition as compared with its P-gp counterparts. Those developed BCRP inhibition hypotheses along with those three models generated in this study are summarized in Table 6. It should be noted that the promiscuous nature of ligand-BCRP interactions can be major hurdles in attempting to establish reliable and predictive *in silico* models. As an example, compound **92** differs from compound **94** in an extra phenyl ring attached to the carbonyl group, whereas their potencies differ by about one order of magnitude, suggesting that the presence of an extra phenyl ring plays a critical role in inhibiting BCRP. On the other hand, Ko143 (**9**), which is a strong BCRP inhibitor, does not possess that extra moiety. Thus, it can exert its potency without aforementioned interaction. As such, different chemical structures can interact with BCRP in different manners. Therefore, it is plausible to expect two different pharmacophore hypotheses adopting different combinations of chemical features will be required to model the inhibition of both types of chemical structures accurately. Otherwise, if only a single theoretical model is employed, substantial predictive errors can be resulted. Indeed, this hypothesis is consistent with the fact that the reported models are severely limited by their global applicability as observed by Pan *et al*. [38].

**Table 6:** Summary of developed BCRP inhibition qualitative and quantitative pharmacophore hypotheses.

		*n*			
Model type	Training	Test	External	Chemical features	Reference
Qualitative					
*HipHop*	4			3 HBAs, 3 HPs	[31]
*HipHop*	23			2 HBAs, 1 HP, 1 RA	[32]
*HipHop*	29			1 HBA, 2 HPs	[33]
*HipHop*	30	79		1 HBA, 3 HPs	[38]
*LigandScout*	4			3 HBAs, 2 HP, 1 RA	[37]
*QuaSAR*	15			1 HBA, 2 RAs	[39]
*Pharmacophore*	90	22	27	1 HBA, 1 HP, 2 RAs	[34]
*Elucidator*					
Quantitative					
*HypoGen* (Hypo A)	22	97	16	1 HBA, 1 HP, 2 RAs	This study
*HypoGen* (Hypo B)	22	97	16	2 HBAs, 1 HP, 1 RA	This study
*HypoGen* (Hypo C)	22	97	16	2 HPs, 2 RAs	This study

Accordingly, once different training samples are selected, different predictive models can be produced. In fact, those published qualitative models by the common features algorithm [31]–[34], [37]–[39] comprised different combinations of chemical features. Even the same molecule in different combinations of training samples can generate different predictive models. As an example, Ko143 was a common molecule in the model development by Matsson *et al*. [33] and Pan *et al*. [38], which selected 1 HBA and 2 HPs as well as 1 HBA and 3 HPs, respectively. Thus, the derived predictive models heavily depend on the samples selected in the training set, suggesting the promiscuous nature of BCRP protein.

Furthermore, a fixed set of training samples do not always guarantee to produce a single consistent model. Four pharmacophore models with different orientations of the same chemical features were derived by Pan *et al*. [38] by use of the same data set. When mapped onto Ko143, the chemical feature HBA coincided with the carbonyl group of the 1,1-dimethylethyl ester and the oxygen atom of the dioxopyrazino ring by 3 and 1 pharmacophore hypotheses, respectively, as demonstrated by Figure 3 of Pan *et al*. [38]. Therefore, it is possible that the same inhibitor can interact with BCRP using different orientations as in the case of hPXR, in which the ligand SR12813 can adopt three different orientations to form cocomplex with protein while the protein conformation remains intact [73]. As a result, the possible multiple ligand orientations imply the promiscuous nature of ligand binding by BCRP. When subjected to validation by the samples in the test set, these four theoretical models showed reasonable performances possibly due to the restriction in model development as well as diverse training samples.

The discrepancies among published qualitative models are consistent with differences among the three quantitative pharmacophore hypothesis derived in this study. For instance, most of developed models employed 1 or 2 HBAs except the one proposed by Chang *et al*. [31], which enlisted 3 HBAs based on a very small number of inhibitors (*n* = 4). Such discrepancies in the number of HBA selection can also be observed from Hypo A and Hypo B, which recruited 1 and 2 HBAs, respectively. Conversely, Hypo C did not consist of any HBA, which is seemingly unusual as compared with published models. Nicolle *et al*., nevertheless, suggested that the HBA feature is not essential for flavonoid-like inhibitors [40].

The predictive model proposed by Sim *et al*. [34] and Hypo A selected 1 HP and 2 RAs that are seemingly inconsistent with the models derived by Chang *et al.* [31] and Pan *et al.* [38], which included 3 HPs. Nevertheless, all 4 models can have the same number of hydrophobic moieties when the two RA features are replaced by the HP groups as suggested by Sim *et al*. [34], giving rise to totally 3 HP features. Also, once the replacement of RA by HP takes place, the predictive models built by Cramer *et al*. [32] and Hypo B, composing of exactly 1 HP and 1 RA features, can have the identical number of hydrophobic moieties with the ones reported by Matsson *et al*. (2 HPs) [33] and Sim *et al*. (2 RAs) [39]. Thus, it can be concluded that those pharmacophore hypotheses in the ensemble can justify the discrepancies among those published models qualitatively.

**Figure 11:**
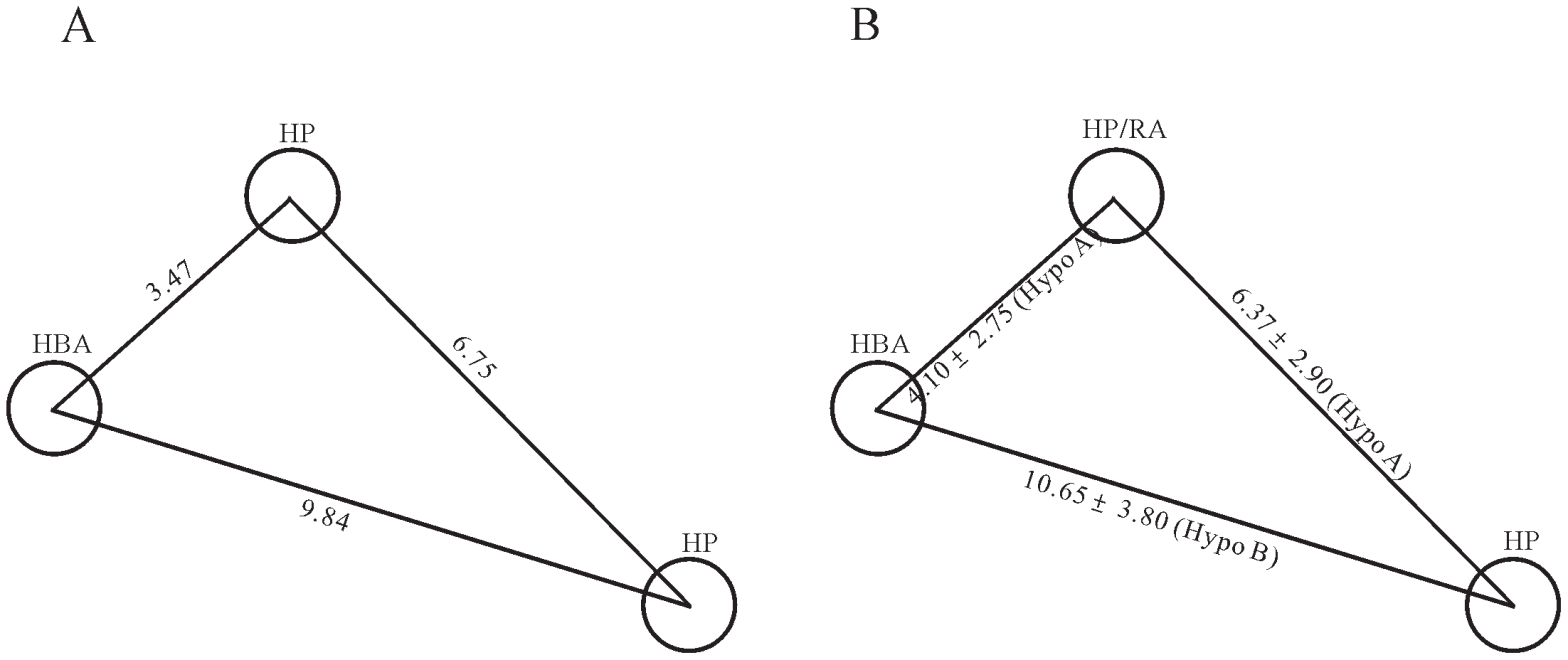
Model proposed by Matsson *et al*. and excerpted model of this study. Geometrical relationships in the pharmacophore models (A) proposed by Matsson *et al*. and (B) excerpted from the PhE in this study. The interfeature distances are measured in Ångstroms.

It has been demonstrated by Sim *et al*. [34] that parts of their model resembled to the model proposed by Matsson *et al*. [33] once the RA feature in their model was replaced by HP and the HP feature in their model was neglected as shown in Figure 4 of Sim *et al*. [34]. However, such geometric constraints seemingly cannot be found from any pharmacophore models in the ensemble. Once those chemical features adopted by those pharmacophore models in the ensemble are taken into account individually, this quantitative inconsistency between PhE and published models can be resolved. For instance, the distance between both HPs in the model built by Matsson *et al*. [33] is 6.75 Å as shown in Figure 11A, which is in good agreement with that between an HP and an RA in Hypo A (6.37±2.90 Å) as illustrated in Figure 11B. The marginal difference between both lengths can be attributed to the tolerances associated with each chemical feature, *viz*. the radius of each sphere. Similarly, the distances between 1 HBA and 2 HPs in their model are 3.47 Å and 9.84 Å, which are in the close ranges of the interfeature distances excerpted from Hypo A (4.10±2.75 Å) and Hypo B (10.65±3.80 Å). When the chemical features associated with each pharmacophore model were treated in an integrated manner, none of pharmacophore models in the ensemble showed significantly quantitative similarity to the model proposed by Matsson *et al*. [33]. Conversely, once those chemical features were treated separately, high levels of quantitative resemblance between the model reported by Matsson *et al.* [33] and those pharmacophore models in the ensemble were yielded.

**Figure 12:**
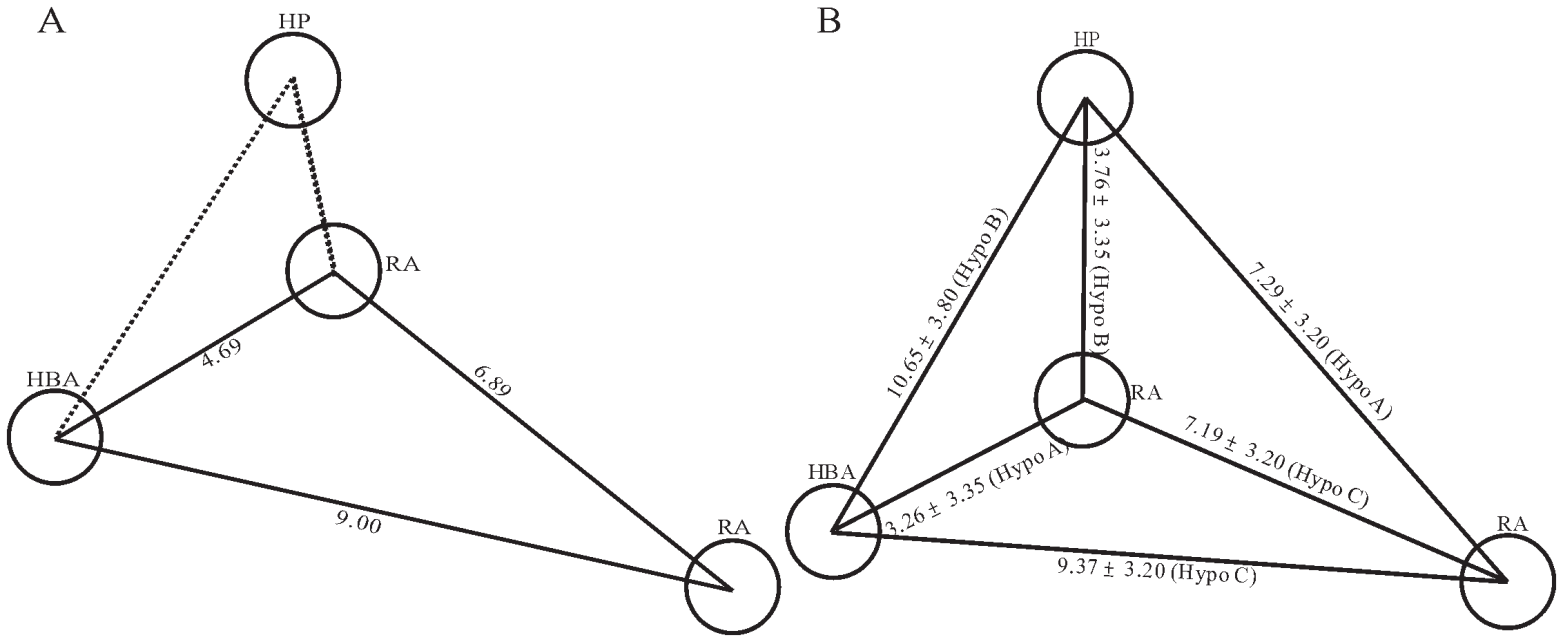
Model proposed by Sim *et al*. and excerpted model of this study. Geometrical relationships in the pharmacophore models (A) proposed by Sim *et al*. and (B) excerpted from the PhE in this study. The interfeature distances are measured in Ångstroms.

The similar observations can also be found in the comparisons between the model reported by Sim *et al*. [34] and those models in the ensemble. The distance between 2 RAs is 6.89 Å in their model (Figure 12A), which is in close range of 7.19±3.20 Å found in Hypo C (Figure 12B). In addition, the interfeature lengths between 1 HBA and 2 RAs are 4.69 Å and 9.00 Å in their model, which, in fact, correspond to the counterparts found in Hypo A (3.26±3.35 Å) and Hypo C (9.37±3.20 Å), respectively. Accordingly, the predictive model built by Sim *et al*. [34] can be reproduced by excerpting some of chemical features from the ensemble. Thus, it can be asserted that PhE/SVM can clearly justify the discrepancies among published models qualitatively and quantitatively.

Consequently, all of those molecules were accurately predicted by PhE/SVM (Table S1), suggesting that PhE/SVM can be applied to a wide range of structurally diverse inhibitors without significant deviations due to the fact that it can properly take into account the promiscuous nature of BCRP as well as the possible ligand orientations. These are normally difficult to be achieved by convention pharmacophore-based modeling techniques.

## Conclusion

BCRP inhibition is critical for drug pharmacokinetics and pharmacodynamics profiling due to its profound involvement in drug-drug interactions as well as its potential influence on clinical efficacy. A theoretical model that can accurately and rapidly predict the inhibition of BCRP can greatly facilitate and expedite the drug discovery and development accordingly. An *in silico* model was derived to quantitatively predict the inhibition of BCRP based on the pharmacophore ensemble/support vector machine scheme to properly address the promiscuous nature of BCRP, which otherwise cannot be reliably modeled by any other analogue-based molecular modeling schemes, when applied to structurally distinct inhibitors. The predictions by the PhE/SVM model are in good agreement with the observed values for structurally diverse 22 and 97 molecules in the training set and test set, respectively. In addition, its robustness was further verified by a group of 16 outliers, which were structurally different from those in the training set. The accuracy and predictivity were assured by a variety of rigorous statistical assessments. When mock tested by a group of HIV PIs to mimic the real challenge, the PhE/SVM model also executed equally well. Furthermore, this theoretical model is able to justify the differences in hitherto reported pharmacophore hypotheses qualitatively and quantitatively. Thus, it can be assured that this PhE/SVM model is an accurate, predictive, and rapid tool that can be employed to facilitate and expedite the drug discovery and development.

## Supporting Information

File S1 Three pharmacophore hypotheses Hypo A, Hypo B, and Hypo C.
(ZIP)

Table S1 Selected compounds for this study, their names, SMILES strings, observed pIC50 values and predicted values by Hypo A, Hypo B, Hypo C, and PhE/SVM, data partitions and references.
(PDF)

## References

[1] Klaassen CD, Aleksunes LM (2010) Xenobiotic, Bile Acid, and Cholesterol Transporters: Function and Regulation. Pharmacol Rev 62: 1–96. 20103563 10.1124/pr.109.002014 PMC2835398

[2] Polgar O, Robey RW, Bates SE (2008) ABCG2: structure, function and role in drug response. Expert Opin Drug Metab Toxicol 4: 1–15. 18370855 10.1517/17425255.4.1.1

[3] Szakács G, Váradi A, Özvegy-Laczka C, Sarkadi B (2008) The role of ABC transporters in drug absorption, distribution, metabolism, excretion and toxicity (ADME-Tox). Drug Discov Today 13: 379–393. 18468555 10.1016/j.drudis.2007.12.010

[4] Vähäkangas K, Myllynen P (2009) Drug transporters in the human blood-placental barrier. Br J Pharmacol 158: 665–678. 19788499 10.1111/j.1476-5381.2009.00336.x PMC2765588

[5] Reyes CL, Chang G (2005) Structure of the ABC Transporter MsbA in Complex with ADP·Vanadate and Lipopolysaccharide. Science 308: 1028–1031. 15890884 10.1126/science.1107733

[6] Chang G (2003) Multidrug resistance ABC transporters. FEBS Lett 555: 102–105. 14630327 10.1016/s0014-5793(03)01085-8

[7] Mao Q (2005) Role of the breast cancer resistance protein (ABCG2) in drug transport. AAPS J 7: E118–E133. 16146333 10.1208/aapsj070112 PMC2751502

[8] Allikmets R, Schriml LM, Hutchinson A, Romano-Spica V, Dean M (1998) A Human Placenta-specific ATP-Binding Cassette Gene (*ABCP*) on Chromosome 4q22 That Is Involved in Multidrug Resistance. Cancer Res 58: 5337–5339. 9850061

[9] Litman T, Druley TE, Stein WD, Bates SE (2001) From MDR to MXR: new understanding of multidrug resistance systems, their properties and clinical significance. Cell Mol Life Sci 58: 931–959. 11497241 10.1007/PL00000912 PMC11337370

[10] Hou X, Huang F, Carboni JM, Flatten K, Asmann YW, et al (2011) Drug Efflux by Breast Cancer Resistance Protein Is a Mechanism of Resistance to the Benzimidazole Insulin-Like Growth Factor Receptor/Insulin Receptor Inhibitor, BMS-536924. Mol Cancer Ther 10: 117–125. 21220496 10.1158/1535-7163.MCT-10-0438 PMC3057506

[11] Shi Z, Tiwari AK, Shukla S, Robey RW, Singh S, et al (2011) Sildenafil Reverses ABCB1- and ABCG2-Mediated Chemotherapeutic Drug Resistance. Cancer Res 71: 3029–3041. 21402712 10.1158/0008-5472.CAN-10-3820 PMC3078184

[12] Muenster U, Grieshop B, Ickenroth K, Gnoth M (2008) Characterization of Substrates and Inhibitors for the In Vitro Assessment of Bcrp Mediated Drug–Drug Interactions. Pharm Res 25: 2320–2326. 18523872 10.1007/s11095-008-9632-1

[13] Hu M, To KKW, Mak VWL, Tomlinson B (2011) The ABCG2 transporter and its relations with the pharmacokinetics, drug interaction and lipid-lowering effects of statins. Expert Opin Drug Metab Toxicol 7: 49–62. 21091277 10.1517/17425255.2011.538383

[14] Hu M, Tomlinson B (2013) Evaluation of the pharmacokinetics and drug interactions of the two recently developed statins, rosuvastatin and pitavastatin. Expert Opin Drug Metab Toxicol 14: 1283–1294. 10.1517/17425255.2014.851667 24156555

[15] Tomlinson B, Hu M, Lee VWY, Lui SSH, Chu TTW, et al (2010) ABCG2 Polymorphism Is Associated With the Low-Density Lipoprotein Cholesterol Response to Rosuvastatin. Clin Pharmacol Ther 87: 558–562. 20130569 10.1038/clpt.2009.232

[16] Declèves X, Jacob A, Yousif S, Shawahna R, Potin S, et al (2011) Interplay of Drug Metabolizing CYP450 Enzymes and ABC Transporters in the Blood-Brain Barrier. Curr Drug Metab 12: 732–741. 21623707 10.2174/138920011798357024

[17] Ciardiello F, Tortora G (2008) EGFR Antagonists in Cancer TreatmentDrug Therapy. N Engl J Med 358: 1160–1174. 18337605 10.1056/NEJMra0707704

[18] Siegel R, Naishadham D, Jemal A (2012) Cancer statistics, 2012. CA Cancer J Clin 62: 10–29. 22237781 10.3322/caac.20138

[19] Elmeliegy MA, Carcaboso AM, Tagen M, Bai F, Stewart CF (2011) Role of ATP-Binding Cassette and Solute Carrier Transporters in Erlotinib CNS Penetration and Intracellular Accumulation. Clin Cancer Res 17: 89–99. 21088257 10.1158/1078-0432.CCR-10-1934 PMC3017236

[20] Gerstner ER, Fine RL (2007) Increased Permeability of the Blood-Brain Barrier to Chemotherapy in Metastatic Brain Tumors: Establishing a Treatment Paradigm. J Clin Oncol 25: 2306–2312. 17538177 10.1200/JCO.2006.10.0677

[21] El Kamar FG, Jindal K, Grossbard ML, Mizrachi HH, Kozuch PS (2004) Pancreatic carcinoma with brain metastases: case report and literature review. Dig Liver Dis 36: 355–360. 15191206 10.1016/j.dld.2003.10.019

[22] Oh Y, Taylor S, Bekele BN, Debnam JM, Allen PK, et al (2009) Number of metastatic sites is a strong predictor of survival in patients with nonsmall cell lung cancer with or without brain metastases. Cancer 115: 2930–2938. 19441110 10.1002/cncr.24333

[23] Huse JT, Holland EC (2010) Targeting brain cancer: advances in the molecular pathology of malignant glioma and medulloblastoma. Nat Rev Cancer 10: 319–331. 20414201 10.1038/nrc2818

[24] Agarwal S, Hartz AMS, Elmquist WF, Bauer B (2011) Breast Cancer Resistance Protein and P-Glycoprotein in Brain Cancer: Two Gatekeepers Team Up. Curr Pharm Des 17: 2793–2802. 21827403 10.2174/138161211797440186 PMC3269897

[25] Schnepf R, Zolk O (2013) Effect of the ATP-binding cassette transporter ABCG2 on pharmacokinetics: experimental findings and clinical implications. Expert Opin Drug Metab Toxicol 9: 287–306. 23289909 10.1517/17425255.2013.742063

[26] Lin JH, Yamazaki M (2003) Role of P-Glycoprotein in Pharmacokinetics: Clinical Implications. Clin Pharmacokinet 42: 59–98. 12489979 10.2165/00003088-200342010-00003

[27] Shukla S, Wu C-P, Ambudkar SV (2008) Development of inhibitors of ATP-binding cassette drug transporters – present status and challenges. Expert Opin Drug Metab Toxicol 4: 205–223. 18248313 10.1517/17425255.4.2.205

[28] Szakács G, Paterson JK, Ludwig JA, Booth-Genthe C, Gottesman MM (2006) Targeting multidrug resistance in cancer. Nat Rev Drug Discov 5: 219–234. 16518375 10.1038/nrd1984

[29] Poguntke M, Hazai E, Fromm MF, Zolk O (2010) Drug transport by breast cancer resistance protein. Expert Opin Drug Metab Toxicol 6: 1363–1384. 20873966 10.1517/17425255.2010.519700

[30] Lagorce D, Reynes C, Camproux A-C, Miteva MA, Sperandio O, et al. (2011) *In Silico* ADME/Tox Predictions. In: Tsaioun K, Kates SA, editors. ADMET for Medicinal Chemists: A Practical Guide. Hoboken, New Jersey: John Wiley & Sons, Inc. pp. 29–124.

[31] Chang C, Ekins S, Bahadduri P, Swaan PW (2006) Pharmacophore-based discovery of ligands for drug transporters. Adv Drug Deliv Rev 58: 1431–1450. 17097188 10.1016/j.addr.2006.09.006 PMC1773055

[32] Cramer J, Kopp S, Bates SE, Chiba P, Ecker GF (2007) Multispecificity of Drug Transporters: Probing Inhibitor Selectivity for the Human Drug Efflux Transporters ABCB1 and ABCG2. ChemMedChem 2: 1783–1788. 17994597 10.1002/cmdc.200700160

[33] Matsson P, Englund G, Ahlin G, Bergström CAS, Norinder U, et al (2007) A Global Drug Inhibition Pattern for the Human ATP-Binding Cassette Transporter Breast Cancer Resistance Protein (ABCG2). J Pharmacol Exp Ther 323: 19–30. 17616561 10.1124/jpet.107.124768

[34] Sim HM, Loh KY, Yeo WK, Lee CY, Go ML (2011) Aurones as Modulators of ABCG2 and ABCB1: Synthesis and Structure–Activity Relationships. ChemMedChem 6: 713–724. 21302361 10.1002/cmdc.201000520

[35] Gandhi Y, Morris M (2009) Structure–Activity Relationships and Quantitative Structure–Activity Relationships for Breast Cancer Resistance Protein (ABCG2). AAPS J 11: 541–552. 19629710 10.1208/s12248-009-9132-1 PMC2758125

[36] Ishikawa T, Hirano H, Saito H, Sano K, Ikegami Y, et al (2012) Quantitative Structure-Activity Relationship (QSAR) Analysis to Predict Drug-Drug Interactions of ABC Transporter ABCG2. Mini Rev Med Chem 12: 505–514. 22587765 10.2174/138955712800493825

[37] Wei Y, Ma Y, Zhao Q, Ren Z, Li Y, et al (2012) New Use for an Old Drug: Inhibiting ABCG2 with Sorafenib. Mol Cancer Ther 11: 1693–1702. 22593228 10.1158/1535-7163.MCT-12-0215

[38] Pan Y, Chothe PP, Swaan PW (2013) Identification of Novel BCRP Inhibitors by Virtual Screening. Mol Pharmaceutics 10: 1236–1248. 10.1021/mp300547h 23418667

[39] Sim H-M, Lee C-Y, Ee PLR, Go M-L (2008) Dimethoxyaurones: Potent inhibitors of ABCG2 (breast cancer resistance protein). Eur J Pharm Sci 35: 293–306. 18725288 10.1016/j.ejps.2008.07.008

[40] Nicolle E, Boumendjel A, Macalou S, Genoux E, Ahmed-Belkacem A, et al (2009) QSAR analysis and molecular modeling of ABCG2-specific inhibitors. Adv Drug Deliv Rev 1: 34–46. 10.1016/j.addr.2008.10.004 19135106

[41] Tan KP, Wang B, Yang M, Boutros PC, MacAulay J, et al (2010) Aryl Hydrocarbon Receptor Is a Transcriptional Activator of the Human Breast Cancer Resistance Protein (BCRP/ABCG2). Mol Pharmacol 78: 175–185. 20460431 10.1124/mol.110.065078

[42] Ecker GF, Stockner T, Chiba P (2008) Computational models for prediction of interactions with ABC-transporters. Drug Discov Today 13: 311–317. 18405843 10.1016/j.drudis.2007.12.012

[43] Leong MK (2007) A Novel Approach Using Pharmacophore Ensemble/Support Vector Machine (PhE/SVM) for Prediction of hERG Liability. Chem Res Toxicol 20: 217–226. 17261034 10.1021/tx060230c

[44] Lexa KW, Carlson HA (2012) Protein flexibility in docking and surface mapping. Q Rev Biophys 45: 301–343. 22569329 10.1017/S0033583512000066 PMC4272345

[45] Leong MK, Chen T-H (2008) Prediction of Cytochrome P450 2B6-Substrate Interactions Using Pharmacophore Ensemble/Support Vector Machine (PhE/SVM) Approach Med Chem. 4: 396–406. 10.2174/157340608784872226 18673154

[46] Leong MK, Chen Y-M, Chen H-B, Chen P-H (2009) Development of a New Predictive Model for Interactions with Human Cytochrome P450 2A6 Using Pharmacophore Ensemble/Support Vector Machine (PhE/SVM) Approach. Pharm Res 26: 987–1000. 19104915 10.1007/s11095-008-9807-9

[47] Chen C-N, Shih Y-H, Ding Y-L, Leong MK (2011) Predicting Activation of the Promiscuous Human Pregnane X Receptor by Pharmacophore Ensemble/Support Vector Machine Approach. Chem Res Toxicol 24: 1765–1778. 21919490 10.1021/tx200310j

[48] Leong MK, Chen H-B, Shih Y-H (2012) Prediction of Promiscuous P-Glycoprotein Inhibition Using a Novel Machine Learning Scheme. PLoS One 7: e33829. 22439003 10.1371/journal.pone.0033829 PMC3306300

[49] Pick A, Müller H, Mayer R, Haenisch B, Pajeva IK, et al (2011) Structure-activity relationships of flavonoids as inhibitors of breast cancer resistance protein (BCRP). Bioorg Med Chem 19: 2090–2102. 21354800 10.1016/j.bmc.2010.12.043

[50] Pick A, Müller H, Wiese M (2008) Structure-activity relationships of new inhibitors of breast cancer resistance protein (ABCG2). Bioorg Med Chem 16: 8224–8236. 18678495 10.1016/j.bmc.2008.07.034

[51] Pick A, Müller H, Wiese M (2009) Novel lead for potent inhibitors of breast cancer resistance protein (BCRP). Bioorg Med Chem Lett 20: 180–183. 19932960 10.1016/j.bmcl.2009.11.004

[52] Juvale K, Pape VFS, Wiese M (2012) Investigation of chalcones and benzochalcones as inhibitors of breast cancer resistance protein. Bioorg Med Chem 20: 346–355. 22112540 10.1016/j.bmc.2011.10.074

[53] Juvale K, Wiese M (2012) 4-Substituted-2-phenylquinazolines as inhibitors of BCRP. Bioorg Med Chem Lett 22: 6766–6769. 23017888 10.1016/j.bmcl.2012.08.024

[54] Chang G, Guida WC, Still CW (1989) An internal-coordinate Monte Carlo method for searching conformational space. J Am Chem Soc 111: 4379–4386.

[55] Kolossváry I, Guida WC (1996) Low Mode Search. An Efficient, Automated Computational Method for Conformational Analysis: Application to Cyclic and Acyclic Alkanes and Cyclic Peptides. J Am Chem Soc 118: 5011–5019.

[56] Still WC, Tempczyk A, Hawley RC, Hendrickson T (1990) Semianalytical treatment of solvation for molecular mechanics and dynamics. J Am Chem Soc 112: 6127–6129.

[57] Halgren TA (1996) Merck molecular force field. I. Basis, form, scope, parameterization, and performance of MMFF94. J Comput Chem 17: 490–519.

[58] Li H, Sutter J, Hoffmann R (2000) HypoGen: An Automated System for Generating 3D Predictive Pharmacophore Models. In: Güner OF, editor. Pharmacophore Perception, Development, and Use in Drug Design. La Jolla, California: International University Line. pp. 171–189.

[59] Evans DA, Doman TN, Thorner DA, Bodkin MJ (2007) 3D QSAR methods: Phase and Catalyst compared. J Chem Inf Model 47: 1248–1257. 17477520 10.1021/ci7000082

[60] Leong MK, Lin S-W, Chen H-B, Tsai F-Y (2010) Predicting Mutagenicity of Aromatic Amines by Various Machine Learning Approaches. Toxicol Sci 116: 498–513. 20507879 10.1093/toxsci/kfq159

[61] Consonni V, Ballabio D, Todeschini R (2010) Evaluation of model predictive ability by external validation techniques. J Chemometr 24: 194–201.

[62] Breiman L, Spector P (1992) Submodel Selection and Evaluation in Regression: The X-Random Case. Int Statist Rev 60: 291–319.

[63] Roy K, Mitra I, Kar S, Ojha PK, Das RN, et al (2012) Comparative Studies on Some Metrics for External Validation of QSPR Models. J Chem Inf Model 52: 396–408. 22201416 10.1021/ci200520g

[64] Golbraikh A, Shen M, Xiao Z, Xiao Y-D, Lee K-H, et al (2003) Rational selection of training and test sets for the development of validated QSAR models. J Comput-Aided Mol Des 17: 241–253. 13677490 10.1023/a:1025386326946

[65] Schüürmann G, Ebert R-U, Chen J, Wang B, Kühne R (2008) External Validation and Prediction Employing the Predictive Squared Correlation Coefficient-Test Set Activity Mean vs Training Set Activity Mean. J Chem Inf Model 48: 2140–2145. 18954136 10.1021/ci800253u

[66] Chirico N, Gramatica P (2012) Real External Predictivity of QSAR Models. Part 2. New Intercomparable Thresholds for Different Validation Criteria and the Need for Scatter Plot Inspection. J Chem Inf Model 52: 2044–2058. 22721530 10.1021/ci300084j

[67] Ojha PK, Mitra I, Das RN, Roy K (2011) Further exploring *r* _m_ ^2^ metrics for validation of QSPR models. Chemometrics Intell Lab Syst 107: 194–205.

[68] Casalegno M, Sello G, Benfenati E (2008) Definition and Detection of Outliers in Chemical Space. J Chem Inf Model 48: 1592–1601. 18652445 10.1021/ci7004065

[69] Reymond J-L, van Deursen R, Blum LC, Ruddigkeit L (2010) Chemical space as a source for new drugs. MedChemComm 1: 30–38.

[70] Gnanadesikan R, Kettenring JR (1972) Robust estimates, residuals, and outlier detection with multiresponse data. Biometrics 28: 81–124.

[71] Weiss J, Rose J, Storch CH, Ketabi-Kiyanvash N, Sauer A, et al (2007) Modulation of human BCRP (ABCG2) activity by anti-HIV drugs. J Antimicrob Chemother 59: 238–245. 17202245 10.1093/jac/dkl474

[72] Gupta A, Zhang Y, Unadkat JD, Mao Q (2004) HIV Protease Inhibitors Are Inhibitors but Not Substrates of the Human Breast Cancer Resistance Protein (BCRP/ABCG2). J Pharmacol Exp Ther 310: 334–341. 15007102 10.1124/jpet.104.065342

[73] Watkins RE, Wisely GB, Moore LB, Collins JL, Lambert MH, et al (2001) The human nuclear xenobiotic receptor PXR: structural determinants of directed promiscuity. Science 292: 2329–2333. 11408620 10.1126/science.1060762

