# Supplementary material for: In Silico Prediction of Inhibition of Promiscuous Breast Cancer Resistance Protein (BCRP/ABCG2)
Source: PLoS One. 2014 Mar 10;9(3):e90689. doi: 10.1371/journal.pone.0090689 (PMC3948701; doi:10.1371/journal.pone.0090689)
Supplement: Table S1 — Selected compounds for this study, their names, SMILES strings, observed pIC50 values and predicted values by Hypo A, Hypo B, Hypo C, and PhE/SVM, data partitions and references. (PDF) [file pone.0090689.s002.pdf]

No.	Molecules	Smile	pIC50(nM)									Set†	References
			Obs.	Hypo A	Δ	Hypo B	Δ	Hypo C	Δ	PhE/SVM	Δ		
**1**	Apigenin	c1(cc(O)c2c(=O)cc(c3ccc(cc3)O)oc2c1)O	5.23	5.62	-0.39	5.04	0.19	4.66	0.57	5.08	0.15	T	BMC 19(2011) 2090-2102
**2**	Ayanin	c1(cc2c(c(c1)O)c(=O)c(OC)c(o2)c1ccc(OC)c(c1)O)OC	6.17	5.60	0.57	6.09	0.08	5.47	0.70	5.88	0.29	T	BMC 19(2011) 2090-2102
**3**	Cyclosporin A	N1(C(CC(C)C)C(N(C(C(N(C(C(NC(C(N(CC(N(C(C(NC(C(N(C(C(NC(C(NC(C(N(C(C1=O)CC(C)C)C)=O)C)=O)C)=O)CC(C)C)C)=O)C(C)C)=O)CC(C)C)C)=O)C)=O)CC)=O)C(O)C(C)C\C=C\C)C)=O)C(C)C)C)=O)C	4.20	3.82	0.38	4.62	-0.42	3.89	0.31	3.93	0.27	T	BMC 16(2008) 8224-8236
**4**	Elacridar	O(C)c1cc2CN(CCc2cc1OC)CCc1ccc(NC(=O)c2c3[nH]c4c(cccc4OC)c(=O)c3ccc2)cc1	6.40	5.51	0.89	5.52	0.88	6.46	-0.06	5.90	0.50	T	BMCL 20(2009) 180-183
**5**	Imatinib	c1(c2nc(Nc3c(C)ccc(c3)NC(=O)c3ccc(cc3)CN3CCN(CC3)C)ncc2)cccnc1	5.35	5.14	0.21	5.15	0.20	5.46	-0.11	5.18	0.17	T	BMC 16(2008) 8224-8236<br>BMCL 20(2009) 180-183
**6**		c1cc(c(cc1OC)O)C(=O)/C=C\c1cc(c(cc1)OC)OC	5.66	5.43	0.23	5.68	-0.02	5.17	0.49	5.48	0.18	T	BMC 20(2012) 346-355
**7**		c1cc(c(c2c1cccc2)O)C(/C=C\c1ccc(Cl)c(c1)Cl)=O	4.59	5.43	-0.85	5.30	-0.72	5.47	-0.88	5.39	-0.80	T	BMC 20(2012) 346-355
**8**		c1cc2c(cc1)nc(nc2Nc1ccc(C(F)(F)F)cc1)c1ccccc1	6.60	6.92	-0.32	5.52	1.08	6.51	0.09	6.61	0.00	T	BMCL 22(2012) 6766-6769
**9**	Ko143	O=C1N2C(c3[nH]c4c(c3CC2C(=O)NC1CCC(OC(C)(C)C)=O)ccc(OC)c4)CC(C)C	6.51	5.62	0.89	6.64	-0.13	5.39	1.12	6.16	0.35	T	BMC 16(2008) 8224-8236<br>BMCL 20(2009) 180-183<br>BMC 19(2011) 2090-2102<br>BMC 20(2012) 346-355<br>BMCL 22(2012) 6766-6769
**10**	Nicardipine	O(C(=O)C=1C(c2cccc([N](=O)O)c2)C(C(OC)=O)=C(NC1C)C)CCN(C)Cc1ccccc1	4.77	5.36	-0.59	5.30	-0.53	4.74	0.02	5.12	-0.35	T	BMC 16(2008) 8224-8236
**11**	Novobiocin	c12c(ccc(c1C)OC1C(O)C(C(C(O1)(C)C)OC)OC(N)=O)c(O)c(c(o2)=O)NC(c1ccc(c(c1)C\C=C(/C)C)O)=O	4.12	5.38	-1.26	5.07	-0.95	4.66	-0.54	4.99	-0.87	T	BMC 16(2008) 8224-8236<br>BMCL 20(2009) 180-183
**12**		c1(c(OC)cc2CN(CCc2c1)CCc1ccc(cc1)NC(=O)c1ccc(cc1)N)OC	3.62	4.12	-0.51	4.01	-0.39	4.66	-1.04	3.97	-0.35	T	BMC 16(2008) 8224-8236
**13**		c1(cc2c(cc1OC)CN(CC2)CCc1ccc(cc1)NC(=O)c1c2c(ncc1)cccc2)OC	4.44	4.60	-0.16	4.47	-0.02	4.66	-0.21	4.37	0.07	T	BMC 16(2008) 8224-8236
**14**		c1(cc2c(cc1OC)CN(CC2)CCc1ccc(cc1)NC(=O)c1nc2c(nc1)cccc2)OC	5.22	4.80	0.43	5.19	0.03	5.47	-0.25	5.05	0.17	T	BMC 16(2008) 8224-8236
**15**		c1(c(OC)cc2CN(CCc2c1)CCc1ccc(NC(c2c(cccc2)[N+]([O-])=O)=O)cc1)OC	4.03	4.12	-0.09	4.43	-0.40	4.66	-0.63	4.16	-0.13	T	BMC 16(2008) 8224-8236
**16**		c1cc2c(cc1)CN(CC2)CCc1ccc(cc1)NC(=O)c1ccccc1[N+]([O-])=O	4.19	4.41	-0.22	4.96	-0.77	4.66	-0.47	4.52	-0.33	T	BMC 16(2008) 8224-8236
**17**		c1(cc2c(cc1OC)CN(CCc1ccc(NC(c3ccccc3N)=O)cc1)CC2)OC	4.23	4.05	0.18	4.00	0.23	4.64	-0.41	3.93	0.30	T	BMC 16(2008) 8224-8236
**18**		c1ccc2CN(CCc3ccc(NC(c4c(cccc4)NC(=O)c4ccc(cc4)[N+](=O)[O-])=O)cc3)CCc2c1	6.16	5.77	0.39	5.27	0.89	5.52	0.64	5.55	0.61	T	BMCL 20(2009) 180-183
**19**	Progesterone	O=C(C1C2(C(CC1)C1C(CC2)C2(CCC(=O)C=C2CC1)C)C)C	3.89	3.80	0.09	3.24	0.65	2.96	0.93	3.19	0.69	T	BMC 16(2008) 8224-8236
**20**	Reserpine	O(C1C(C2C(CN3CCc4c([nH]c5c4ccc(OC)c5)C3C2)CC1OC(=O)c1cc(OC)c(OC)c(OC)c1)C(OC)=O)C	4.59	4.85	-0.27	4.89	-0.30	5.52	-0.94	4.94	-0.35	T	BMC 16(2008) 8224-8236
**21**	Retusin	c1(cc2c(c(c1)O)c(c(c(o2)c1ccc(OC)c(c1)OC)OC)=O)OC	6.05	5.68	0.37	6.01	0.03	5.47	0.58	5.88	0.17	T	BMC 19(2011) 2090-2102
**22**	Tariquidar	O(c1cc2CN(CCc2cc1OC)CCc1ccc(NC(=O)c2c(NC(=O)c3cc4ccccc4nc3)cc(OC)c(OC)c2)cc1)C	5.92	5.89	0.03	5.54	0.38	5.85	0.07	5.86	0.06	T	BMC 19(2011) 2090-2102<br>BMCL 20(2009) 180-183
**23**	6-Methoxyflavanon	c1c(cc2C(CC(Oc2c1)c1ccccc1)=O)OC	4.34	4.16	0.17	4.35	-0.02	4.57	-0.24	4.11	0.22	t	BMC 19(2011) 2090-2102
**24**	6-Methoxyflavone	c1c(cc2c(=O)cc(c3ccccc3)oc2c1)OC	5.00	5.64	-0.64	4.62	0.38	5.37	-0.37	5.11	-0.11	t	BMC 19(2011) 2090-2102
**25**	Calflorenon B	C1C(OC2=C(C1OC)C(C(C1(OC)Oc3c(C21OC)c(c(c1c3C(O)CC(c2ccccc2)O1)OC)OC)OC)=O)c1ccccc1	4.40	4.39	0.00	4.86	-0.47	4.64	-0.24	4.46	-0.06	t	BMC 19(2011) 2090-2102
**26**	Chrysin-dimethylether	c1(cc(OC)c2c(=O)cc(c3ccccc3)oc2c1)OC	4.96	5.64	-0.68	4.85	0.11	5.38	-0.42	5.23	-0.27	t	BMC 19(2011) 2090-2102
**27**	Chysin	c1(cc(O)c2c(=O)cc(c3ccccc3)oc2c1)O	5.59	5.64	-0.06	5.08	0.50	4.66	0.93	5.11	0.47	t	BMC 19(2011) 2090-2102
**28**	Diltiazem	S1C(c2ccc(OC)cc2)C(OC(=O)C)C(=O)N(CCN(C)C)c2c1cccc2	3.07	3.88	-0.81	4.57	-1.50	3.81	-0.74	3.91	-0.84	t	BMC 16(2008) 8224-8236
**29**	Dimethylcryptostrobin	c1(cc(OC)c2C(CC(Oc2c1C)c1ccccc1)=O)OC	4.77	4.77	0.00	4.81	-0.04	4.71	0.06	4.62	0.15	t	BMC 19(2011) 2090-2102
**30**	Dimethylpinocembrin	c1(cc(OC)c2C(CC(Oc2c1)c1ccccc1)=O)OC	4.49	5.03	-0.54	4.58	-0.08	4.55	-0.05	4.57	-0.08	t	BMC 19(2011) 2090-2102
**31**	Flavone	c1ccc2c(=O)cc(c3ccccc3)oc2c1	4.59	5.64	-1.06	4.59	-0.01	4.65	-0.06	4.87	-0.29	t	BMC 19(2011) 2090-2102

**32**	Gefitinib	O1CCN(CC1)CCCOc1cc2c(cc1OC)ncnc2Nc1ccc(c(c1)Cl)F	5.96	5.65	0.30	5.96	0.00	5.71	0.25	5.92	0.04	t	BMCL 20(2009) 180-183
**33**	Genistein	c1(cc2c(c(c1)O)c(c(co2)c1ccc(O)cc1)=O)O	5.06	4.99	0.06	4.92	0.14	4.64	0.41	4.74	0.31	t	BMC 19(2011) 2090-2102
**34**		c1cc(c(cc1O)O)C(=O)/C=C\c1ccccc1Cl	5.49	5.49	0.00	5.43	0.06	4.66	0.83	5.22	0.28	t	BMC 20(2012) 346-355
**35**		c1cc(c(cc1O)O)C(/C=C\c1ccc(Cl)cc1)=O	5.22	5.40	-0.19	5.61	-0.39	5.22	0.00	5.45	-0.23	t	BMC 20(2012) 346-355
**36**		c1cc(c(cc1OC)O)C(/C=C\c1ccccc1)=O	4.51	5.40	-0.90	4.86	-0.35	4.83	-0.32	4.95	-0.44	t	BMC 20(2012) 346-355
**37**		c1cc(c(cc1OC)O)C(/C=C\c1ccc(OC)cc1)=O	5.38	5.40	-0.02	5.61	-0.23	5.38	0.00	5.50	-0.13	t	BMC 20(2012) 346-355
**38**		c1cc(c(cc1OC)O)C(/C=C\c1cc(ccc1)OC)=O	5.50	5.50	0.00	5.49	0.01	5.48	0.02	5.52	-0.02	t	BMC 20(2012) 346-355
**39**		c1cc(c(cc1OC)O)C(=O)/C=C\c1ccccc1Cl	5.16	5.25	-0.08	5.16	0.00	5.19	-0.02	5.14	0.02	t	BMC 20(2012) 346-355
**40**		c1cc(c(cc1OC)O)C(=O)/C=C\c1ccc(cc1)Cl	4.87	5.24	-0.37	5.58	-0.71	4.94	-0.07	5.27	-0.40	t	BMC 20(2012) 346-355
**41**		c1c(OC)cc(c(c1)C(/C=C\c1ccc(OC)cc1)=O)OC	5.63	5.63	0.00	5.63	0.00	5.53	0.10	5.67	-0.04	t	BMC 20(2012) 346-355
**42**		c1cc(c(cc1OC)OC)C(/C=C\c1cc(c(cc1)OC)OC)=O	5.66	5.65	0.01	5.63	0.03	5.54	0.12	5.69	-0.03	t	BMC 20(2012) 346-355
**43**		c1c(OC)cc(c(c1)C(/C=C\c1c(cccc1)Cl)=O)OC	5.50	5.50	0.01	5.16	0.35	5.50	0.00	5.36	0.14	t	BMC 20(2012) 346-355
**44**		c1c(OC)cc(c(c1)C(/C=C\c1ccc(Cl)cc1)=O)OC	5.51	5.49	0.02	5.75	-0.24	5.51	0.00	5.66	-0.15	t	BMC 20(2012) 346-355
**45**		c1c(cc(O)c(C(=O)/C=C\c2ccc(cc2)OC)c1OC)OC	5.23	5.35	-0.11	5.58	-0.35	5.23	0.00	5.41	-0.18	t	BMC 20(2012) 346-355
**46**		c1c(cc(O)c(C(=O)/C=C\c2cc(c(cc2)OC)OC)c1OC)OC	6.12	5.77	0.35	6.12	0.01	5.55	0.58	6.01	0.12	t	BMC 20(2012) 346-355
**47**		c1c(cc(O)c(C(=O)/C=C\c2c(cccc2)Cl)c1OC)OC	5.28	5.33	-0.05	5.28	0.00	5.28	0.00	5.27	0.01	t	BMC 20(2012) 346-355
**48**		c1cc(cc(c1OC)OC)C(=O)/C=C\c1ccc(cc1)OC	5.66	5.65	0.01	5.66	0.00	5.50	0.16	5.69	-0.03	t	BMC 20(2012) 346-355
**49**		c1cc(cc(c1OC)OC)C(=O)/C=C\c1cc(c(cc1)OC)OC	5.48	5.48	0.00	5.48	0.00	5.49	0.00	5.51	-0.03	t	BMC 20(2012) 346-355
**50**		c1c(c(OC)cc(C(=O)/C=C\c2ccccc2Cl)c1)OC	4.96	5.20	-0.25	4.96	0.00	5.14	-0.18	5.01	-0.05	t	BMC 20(2012) 346-355
**51**		c1cc(cc(c1OC)OC)C(=O)/C=C\c1ccc(Cl)cc1	5.57	5.56	0.00	5.57	0.00	5.55	0.01	5.62	-0.05	t	BMC 20(2012) 346-355
**52**		c1cc(c(c2c1cccc2)O)C(/C=C\c1ccccc1)=O	4.61	5.41	-0.79	4.73	-0.11	4.74	-0.12	4.86	-0.25	t	BMC 20(2012) 346-355
**53**		c1c2c(cccc2)c(O)c(C(=O)/C=C\c2ccc(cc2)OC)c1	4.77	5.41	-0.64	5.03	-0.26	5.01	-0.24	5.09	-0.32	t	BMC 20(2012) 346-355
**54**		c1cc(c(c2c1cccc2)O)C(/C=C\c1ccc(OC)c(c1)OC)=O	5.60	5.60	0.00	5.69	-0.09	5.54	0.06	5.69	-0.09	t	BMC 20(2012) 346-355
**55**		c1cc(c(c2c1cccc2)O)C(/C=C\c1cccc(c1)OC)=O	4.92	5.44	-0.51	4.92	0.00	4.92	0.00	5.02	-0.10	t	BMC 20(2012) 346-355
**56**		c12ccccc2c(c(cc1)O)C(/C=C\c1ccccc1)=O	5.56	5.54	0.02	5.24	0.32	5.45	0.11	5.40	0.16	t	BMC 20(2012) 346-355
**57**		c12c(c(cc1)O)C(=O)/C=C\c1ccc(cc1)OC)cccc2	5.42	5.44	-0.02	5.59	-0.17	5.42	0.00	5.53	-0.11	t	BMC 20(2012) 346-355
**58**		c12ccc(O)c(C(=O)/C=C\c3cc(c(cc3)OC)OC)c1cccc2	5.74	5.73	0.00	5.78	-0.05	5.56	0.18	5.81	-0.07	t	BMC 20(2012) 346-355
**59**		c12c(c(cc1)O)C(=O)/C=C\c1cc(ccc1)OC)cccc2	5.71	5.73	-0.01	5.71	0.01	5.54	0.17	5.76	-0.05	t	BMC 20(2012) 346-355
**60**		c12ccccc2c(c(cc1)O)C(/C=C\c1ccccc1Cl)=O	5.28	5.29	0.00	5.25	0.04	5.29	0.00	5.24	0.04	t	BMC 20(2012) 346-355
**61**		c12ccc(O)c(C(=O)/C=C\c3cc(c(cc3)Cl)Cl)c1cccc2	5.06	5.30	-0.24	5.80	-0.74	5.35	-0.29	5.55	-0.49	t	BMC 20(2012) 346-355
**62**		c1cc(c(cc1)O)C(/C=C\c1ccc(OC)c(c1)OC)=O	5.24	5.38	-0.14	5.63	-0.39	5.24	0.00	5.46	-0.22	t	BMC 20(2012) 346-355
**63**		c1c(cc(O)c(C(/C=C\c2ccc(OC)cc2)=O)c1)O	4.48	5.40	-0.92	5.61	-1.13	4.70	-0.22	5.28	-0.80	t	BMC 20(2012) 346-355
**64**		c1c(cc(O)c(C(=O)/C=C\c2ccc(OC)c(c2)OC)c1)O	6.03	5.79	0.24	6.03	0.00	5.42	0.61	5.92	0.11	t	BMC 20(2012) 346-355
**65**		c1cc(c(cc1O)O)C(=O)/C=C\c1cc(ccc1)OC	5.55	5.55	0.00	5.55	0.01	5.38	0.17	5.54	0.01	t	BMC 20(2012) 346-355
**66**		c1cc2c(cc1)nc(c1ccccc1)nc2Nc1cccc(c1)[N+]([O-])=O	5.23	5.72	-0.49	5.25	-0.02	5.90	-0.67	5.65	-0.42	t	BMCL 22(2012) 6766-6769
**67**		c1cc2c(cc1)nc(c1ccccc1)nc2Nc1ccc([N+]([O-])=O)cc1	6.89	6.84	0.04	6.23	0.65	5.93	0.95	6.72	0.16	t	BMCL 22(2012) 6766-6769
**68**		c1ccc2nc(c3ccccc3)nc(c2c1)Nc1cc(c(cc1)F)[N](=O)O	6.01	5.81	0.20	5.95	0.07	5.92	0.09	6.07	-0.06	t	BMCL 22(2012) 6766-6769
**69**		c1cc2c(cc1)nc(c1ccccc1)nc2Nc1cccc(c1)C#N	5.61	5.69	-0.08	6.02	-0.41	5.91	-0.30	6.05	-0.44	t	BMCL 22(2012) 6766-6769
**70**		c1cc2c(cc1)nc(nc2N1CCN(c2ccccc2)CC1)c1ccccc1	5.82	5.82	0.00	5.46	0.35	6.30	-0.48	5.96	-0.14	t	BMCL 22(2012) 6766-6769
**71**		c1cc2c(cc1)nc(nc2N1CCN(c2ccc([N+]([O-])=O)cc2)CC1)c1ccccc1	5.10	5.65	-0.55	5.10	0.00	5.10	0.00	5.27	-0.17	t	BMCL 22(2012) 6766-6769
**72**		c1ccc2nc(c3ccccc3)nc(c2c1)Nc1c(cccc1)Br	5.03	5.65	-0.62	4.20	0.82	4.99	0.04	4.80	0.23	t	BMCL 22(2012) 6766-6769
**73**	Kaempferol	c1(cc2c(c(c1)O)c(c(O)c(o2)c1ccc(cc1)O)=O)O	5.21	5.58	-0.37	5.12	0.09	4.66	0.55	5.10	0.10	t	BMC 19(2011) 2090-2102
**74**	Morin	c1(O)cc2c(c(c1)O)c(c(c(o2)c1ccc(O)cc1O)O)=O	4.31	5.61	-1.30	4.97	-0.66	4.64	-0.33	5.04	-0.73	t	BMC 19(2011) 2090-2102
**75**	Neocalycopteron	C1C(c2ccccc2)OC23C(=C1)C(C(=C(C2(OC)c1c(OC)c(OC)c2OC(c4ccccc4)CC (c2c1O3)O)OC)OC)=O	4.82	4.81	0.01	5.19	-0.36	4.86	-0.03	4.86	-0.04	t	BMC 19(2011) 2090-2102
**76**	Nobiletin	c1(c(OC)c(OC)c2c(=O)cc(c3cc(c(cc3)OC)OC)oc2c1OC)OC	5.36	5.36	0.00	5.42	-0.07	5.48	-0.12	5.42	-0.07	t	BMC 19(2011) 2090-2102
**77**	Penduletin	c1(cc2c(c(c1OC)O)c(=O)c(c(o2)c1ccc(O)cc1)OC)OC	5.70	5.70	0.00	5.10	0.60	5.53	0.17	5.43	0.27	t	BMC 19(2011) 2090-2102
**78**		c1cc2c(cc1)CN(CC2)CCc1ccc(cc1)NC(c1ccccc1N)=O	4.34	4.66	-0.32	4.32	0.02	4.65	-0.31	4.33	0.01	t	BMC 16(2008) 8224-8236
**79**		c1(c(OC)cc2CN(CCc2c1)CCc1ccc(cc1)NC(=O)c1ccc(cc1)[N+]([O-])=O)OC	5.12	5.28	-0.16	5.12	0.00	5.12	0.00	5.11	0.01	t	BMC 16(2008) 8224-8236

**80**		c1ccc2CN(CCc3ccc(cc3)NC(=O)c3ccc([N+](=O)[O-])cc3)CCc2c1	5.17	5.17	0.00	5.17	0.00	5.25	-0.08	5.13	0.04	t	BMC 16(2008) 8224-8236
**81**		c1cc2c(cc1)CN(CCc1ccc(cc1)NC(=O)c1ccc(cc1)N)CC2	4.11	4.70	-0.59	4.12	-0.01	4.65	-0.54	4.25	-0.14	t	BMC 16(2008) 8224-8236
**82**		c1(c(OC)cc2CN(CCc2c1)CCc1ccc(cc1)NC(=O)c1cc2c(nc1)cccc2)OC	4.62	5.57	-0.95	4.62	0.00	5.30	-0.68	5.06	-0.44	t	BMC 16(2008) 8224-8236
**83**		c1ccc2CN(CCc3ccc(cc3)NC(c3cc4c(nc3)cccc4)=O)CCc2c1	5.30	5.57	-0.27	5.31	-0.01	5.30	0.00	5.40	-0.10	t	BMC 16(2008) 8224-8236
**84**		c1(c(OC)cc2CN(CCc3ccc(NC(c4ccc(OC)c(c4)OC)=O)cc3)CCc2c1)OC	4.32	4.82	-0.50	4.92	-0.60	5.35	-1.03	4.89	-0.57	t	BMC 16(2008) 8224-8236
**85**		c1(c(OC)cc2CN(CCc3ccc(NC(c4cc(OC)c(OC)cc4[N+]([O-])=O)=O)cc3)CCc2c1)OC	4.19	4.63	-0.44	5.19	-1.00	5.35	-1.16	4.94	-0.75	t	BMC 16(2008) 8224-8236
**86**		c1(cc2c(cc1OC)CN(CC2)CCc1ccc(cc1)NC(=O)c1cc2c(cc1)OCO2)OC	4.82	4.82	0.00	4.79	0.03	4.82	0.00	4.66	0.16	t	BMC 16(2008) 8224-8236
**87**		c1(cc2c(cc1OC)CN(CC2)CCc1ccc(cc1)NC(=O)c1c(cccc1)Br)OC	4.66	4.75	-0.09	4.66	0.00	4.95	-0.29	4.61	0.05	t	BMC 16(2008) 8224-8236
**88**		c1(c(OC)cc2CN(CCc3ccc(cc3)NC(=O)c3cc(ccc3)Br)CCc2c1)OC	4.64	4.66	-0.02	4.64	0.00	5.14	-0.50	4.62	0.02	t	BMC 16(2008) 8224-8236
**89**		c1(c(OC)cc2CN(CCc2c1)CCc1ccc(cc1)NC(=O)c1ccc(cc1)Br)OC	4.94	4.94	0.00	4.94	0.00	5.55	-0.61	5.01	-0.07	t	BMC 16(2008) 8224-8236
**90**		c1(c(cc2CN(CCc2c1)CCc1ccc(cc1)NC(=O)c1ccccc1)OC)OC	4.61	4.75	-0.14	4.61	0.00	4.73	-0.12	4.52	0.09	t	BMC 16(2008) 8224-8236
**91**		c1(cc2c(cc1OC)CN(CC2)CCc1ccc(cc1)NC(=O)c1c2c(ccc1)cccc2)OC	4.89	4.93	-0.04	4.89	0.00	4.90	-0.01	4.78	0.11	t	BMC 16(2008) 8224-8236
**92**		c1(cc2c(cc1OC)CN(CC2)CCc1ccc(cc1)NC(=O)c1cc2c(cc1)cccc2)OC	6.14	5.63	0.51	5.70	0.44	5.53	0.61	5.71	0.43	t	BMC 16(2008) 8224-8236
**93**		c1(c(OC)cc2CN(CCc2c1)CCc1ccc(cc1)NC(=O)c1cc2c(cn1)cccc2)OC	5.22	5.46	-0.24	5.22	0.00	5.45	-0.23	5.36	-0.14	t	BMC 16(2008) 8224-8236
**94**		c1(c(cc2CN(CCc2c1)CCc1ccc(cc1)NC(=O)c1cccnc1)OC)OC	4.35	4.76	-0.41	4.63	-0.28	4.80	-0.45	4.55	-0.20	t	BMC 16(2008) 8224-8236
**95**		c1(cc2c(cc1OC)CN(CC2)CCc1ccc(cc1)NC(=O)c1cc2c(cc1)ccnc2)OC	4.84	5.39	-0.55	4.84	0.00	5.22	-0.38	5.06	-0.22	t	BMC 16(2008) 8224-8236
**96**		c1(cc2c(cc1OC)CN(CC2)CCc1ccc(cc1)NC(=O)c1ccccc1NC(c1ccc(OC)c(OC)c1)=O)OC	5.82	5.88	-0.06	5.83	-0.01	6.21	-0.39	6.15	-0.33	t	BMC 16(2008) 8224-8236
**97**		c1cc2c(cc1)CN(CC2)CCc1ccc(NC(Nc2c([N+](=O)[O-])cccc2)=O)cc1	5.17	5.38	-0.21	5.17	0.00	5.14	0.03	5.19	-0.02	t	BMCL 20(2009) 180-183
**98**		c1(cc2c(cc1OC)CN(CCc1ccc(NC(Nc3c([N+](=O)[O-])cccc3)=O)cc1)CC2)OC	4.99	5.40	-0.41	5.15	-0.16	4.99	0.00	5.14	-0.15	t	BMCL 20(2009) 180-183
**99**		c1(OC)c(OC)cc2CN(CCc2c1)CCc1ccc(cc1)NC(=O)Nc1ccc(cc1)[N+](=O)[O-]	5.41	5.40	0.01	5.40	0.01	5.42	-0.01	5.41	0.00	t	BMCL 20(2009) 180-183
**100**		c1cc2c(cc1)CN(CC2)CCc1ccc(cc1)NC(=O)Nc1c(N)cccc1	3.99	5.23	-1.23	4.00	-0.01	4.54	-0.55	4.39	-0.40	t	BMCL 20(2009) 180-183
**101**		c1(cc2c(cc1OC)CN(CCc1ccc(NC(Nc3c(N)cccc3)=O)cc1)CC2)OC	4.18	5.34	-1.16	4.18	0.00	4.70	-0.52	4.57	-0.39	t	BMCL 20(2009) 180-183
**102**		c1(OC)c(OC)cc2CN(CCc2c1)CCc1ccc(cc1)NC(=O)Nc1ccc(cc1)N	4.13	5.33	-1.20	4.13	0.00	4.71	-0.58	4.54	-0.41	t	BMCL 20(2009) 180-183
**103**		c1(cc2c(cc1OC)CN(CCc1ccc(NC(Nc3c(NC(=O)c4ccc(OC)c(c4)OC)cccc3)=O)cc1)CC2)OC	4.99	5.59	-0.60	5.11	-0.11	5.80	-0.81	5.48	-0.49	t	BMCL 20(2009) 180-183
**104**		c1cc2c(cc1)CN(CC2)CCc1ccc(cc1)NC(c1c(cccc1)NC(Nc1ccc([N+]([O-])=O)cc1)=O)=O	6.27	6.25	0.02	6.27	-0.01	6.24	0.03	6.58	-0.31	t	BMCL 20(2009) 180-183
**105**		c1(c(c2c(c(c1OC)OC)c(=O)c(c(o2)c1ccc(OC)c(c1)OC)OC)OC)OC	5.80	5.68	0.12	5.80	-0.01	5.56	0.24	5.80	0.00	t	BMC 19(2011) 2090-2102
**106**		c1(c(c(O)c2c(=O)cc(c3ccccc3)oc2c1)C)OC	5.55	5.66	-0.11	4.98	0.57	5.49	0.06	5.34	0.21	t	BMC 19(2011) 2090-2102
**107**		c1(c(C)c(OC)c2c(=O)cc(c3ccccc3)oc2c1)OC	5.10	5.66	-0.56	4.96	0.14	5.42	-0.32	5.31	-0.21	t	BMC 19(2011) 2090-2102
**108**		C1C(c2ccccc2)OC23C(=C1)C(C(=C(C2(OC)c1c(OC)c(OC)c2OC(c4ccccc4)CC(c2c1O3)OC)OC)OC)=O	4.33	4.72	-0.39	4.95	-0.62	4.90	-0.57	4.72	-0.39	t	BMC 19(2011) 2090-2102
**109**	Pinostrombin	c1(cc(O)c2C(CC(Oc2c1)c1ccccc1)=O)OC	4.51	5.20	-0.69	4.43	0.08	4.53	-0.02	4.57	-0.06	t	BMC 19(2011) 2090-2102
**110**	Quercetin	c1(cc2c(c(c1)O)c(c(O)c(o2)c1ccc(c(c1)O)O)=O)O	5.12	5.49	-0.37	5.12	0.00	4.66	0.46	5.06	0.05	t	BMC 19(2011) 2090-2102
**111**	Sinensetin	c1(c(c(OC)c2c(=O)cc(c3cc(c(cc3)OC)OC)oc2c1)OC)OC	5.08	5.39	-0.31	5.41	-0.33	5.47	-0.39	5.43	-0.35	t	BMC 19(2011) 2090-2102
**112**	Strobopinin	c1(c(c(O)c2C(CC(Oc2c1)c1ccccc1)=O)C)OC	4.59	4.55	0.03	4.75	-0.17	4.54	0.04	4.45	0.14	t	BMC 19(2011) 2090-2102
**113**	Strobopinin-7-methylether	c1(c(c(O)c2C(CC(Oc2c1)c1ccccc1)=O)C)O	4.92	4.94	-0.02	4.92	0.00	4.96	-0.04	4.82	0.11	t	BMC 19(2011) 2090-2102
**114**	Tangeretin	c1(c(OC)c(OC)c2c(=O)cc(c3ccc(cc3)OC)oc2c1OC)OC	4.77	5.50	-0.73	5.41	-0.64	5.49	-0.72	5.48	-0.71	t	BMC 19(2011) 2090-2102
**115**	Verapamil	O(c1cc(C(CCCN(C)CCc2ccc(OC)c(OC)c2)(C(C)C)C#N)ccc1OC)C	3.28	3.75	-0.47	4.66	-1.38	4.83	-1.55	4.16	-0.88	t	BMC 16(2008) 8224-8236
**116**	XR9456	c1(cc2c(cc1OC)CN(CC2)CCc1ccc(cc1)NC(=O)c1ccccc1NC(c1ccccc1)=O)OC	5.40	5.95	-0.55	5.28	0.12	5.76	-0.36	5.72	-0.32	t	BMC 16(2008) 8224-8236
**117**	XR9504	c1(cc2c(cc1OC)CN(CCc1ccc(cc1)NC(c1c(NC(=O)c3ccc(cc3)C)cccc1)=O)CC2)OC	5.41	5.93	-0.52	5.75	-0.34	5.88	-0.47	6.00	-0.59	t	BMC 16(2008) 8224-8236
**118**	XR9544	c1(cc2c(cc1OC)CN(CC2)CCc1ccc(NC(c2c(NC(=O)c3cnc4c(c3)cccc4)cccc2)=O)cc1)OC	5.30	6.18	-0.88	5.32	-0.02	5.83	-0.53	5.88	-0.58	t	BMC 16(2008) 8224-8236
**119**	XR9577	c1ccc2CN(CCc3ccc(NC(c4c(cccc4)NC(=O)c4cnc5c(c4)cccc5)=O)cc3)CCc2c1	6.00	6.25	-0.25	6.00	0.00	6.00	0.00	6.34	-0.34	t	BMC 16(2008) 8224-8236

**120**	c1cc2c(cc1)nc(nc2Nc1ccc(OC)c(c1)OC)c1ccccc1	5.59	5.74	-0.15	4.51	1.08	5.91	-0.32	5.29	0.30	o	BMCL 22(2012) 6766-6769
**121**	c1ccc2nc(c3ccccc3)nc(c2c1)Nc1c(cccc1)[N](O)=O	6.72	5.68	1.05	4.52	2.20	5.90	0.82	5.26	1.46	o	BMCL 22(2012) 6766-6769
**122**	c1cc2c(cc1)nc(nc2Nc1cccc(c1)C(F)(F)F)c1ccccc1	6.85	5.72	1.13	5.21	1.65	6.18	0.67	5.73	1.12	o	BMCL 22(2012) 6766-6769
**123**	c1cc2c(cc1)nc(nc2N1CCN(c2cccc(Br)c2)CC1)c1ccccc1	5.05	5.70	-0.65	5.31	-0.26	6.42	-1.37	5.87	-0.82	o	BMCL 22(2012) 6766-6769
**124**	c1ccc2nc(c3ccccc3)nc(c2c1)Nc1ccccc1	5.29	5.72	-0.43	5.27	0.02	5.88	-0.59	5.65	-0.36	o	BMCL 22(2012) 6766-6769
**125**	c1cc2c(cc1)nc(nc2N1CCN(c2cccc(Cl)c2)CC1)c1ccccc1	5.43	5.74	-0.32	5.26	0.16	5.88	-0.45	5.66	-0.23	o	BMCL 22(2012) 6766-6769
**126**	c1cc2c(cc1)nc(nc2N1CCN(c2ccc(F)c(Cl)c2)CC1)c1ccccc1	5.23	5.67	-0.44	4.96	0.27	5.85	-0.62	5.46	-0.23	o	BMCL 22(2012) 6766-6769
**127**	c1cc2c(cc1)nc(nc2N1CCN(c2cccc(OC)c2)CC1)c1ccccc1	5.09	5.73	-0.65	5.33	-0.25	5.91	-0.83	5.70	-0.61	o	BMCL 22(2012) 6766-6769
**128**	c1cc2c(cc1)nc(nc2N1CCN(c2cccc(c2)[N+]([O-])=O)CC1)c1ccccc1	5.05	5.73	-0.68	5.38	-0.33	6.28	-1.23	5.86	-0.81	o	BMCL 22(2012) 6766-6769
**129**	c1ccc2nc(c3ccccc3)nc(c2c1)Nc1cccc(c1)Br	6.24	6.81	-0.57	6.19	0.05	6.72	-0.48	7.00	-0.76	o	BMCL 22(2012) 6766-6769
**130**	c1cc2c(cc1)nc(nc2Nc1ccc(Br)cc1)c1ccccc1	5.17	7.09	-1.92	5.66	-0.49	5.92	-0.76	6.53	-1.37	o	BMCL 22(2012) 6766-6769
**131**	c1cc2c(cc1)nc(c1ccccc1)nc2Nc1cccc(c1)Cl	5.71	5.65	0.06	4.17	1.55	5.05	0.66	4.80	0.91	o	BMCL 22(2012) 6766-6769
**132**	c1cc2c(cc1)nc(c1ccccc1)nc2Nc1ccc(F)c(c1)Cl	5.78	5.64	0.14	5.13	0.65	5.30	0.48	5.34	0.44	o	BMCL 22(2012) 6766-6769
**133**	c1cc2c(cc1)nc(nc2Nc1ccccc1OC)c1ccccc1	5.65	5.65	0.00	5.13	0.52	5.18	0.46	5.31	0.34	o	BMCL 22(2012) 6766-6769
**134**	c1ccc2nc(c3ccccc3)nc(c2c1)Nc1cc(ccc1)OC	5.88	5.64	0.24	5.10	0.78	5.09	0.79	5.26	0.62	o	BMCL 22(2012) 6766-6769
**135**	c1ccc2nc(c3ccccc3)nc(c2c1)Nc1ccc(cc1)OC	5.71	5.65	0.07	5.12	0.60	5.06	0.65	5.26	0.45	o	BMCL 22(2012) 6766-6769

†T, t, and o stand for the training set, test set, and outlier set, respectively.
